# Adaptive predator prey algorithm for many objective optimization

**DOI:** 10.1038/s41598-025-96901-y

**Published:** 2025-04-12

**Authors:** Nikunj Mashru, Kanak Kalita, Lenka Čepová, Pinank Patel, Arpita, Pradeep Jangir

**Affiliations:** 1 Department of Mechanical Engineering, Marwadi University, Rajkot, 360003 India; 2 Department of Mechanical Engineering, Vel Tech Rangarajan Dr. Sagunthala R&D Institute of Science and Technology, Avadi, 600062 India; 3 Department of Machining, Assembly and Engineering Metrology, Faculty of Mechanical Engineering, VSB-Technical University of Ostrava, 70800 Ostrava, Czech Republic; 4 Department of Biosciences, Saveetha School of Engineering, Saveetha Institute of Medical and Technical Sciences, Chennai, 602105 India; 5 University Centre for Research and Development, Chandigarh University, Mohali, 140413 India; 6 Department of CSE, Graphic Era Hill University, Dehradun, 248002 India; 7 Department of CSE, Graphic Era Deemed to be University, Dehradun, Uttarakhand 248002 India; 8 Applied Science Research Center, Applied Science Private University, Amman, 11931 Jordan; 9 Centre for Research Impact & Outcome, Chitkara University Institute of Engineering and Technology, Chitkara University, Rajpura, Punjab 140401 India

**Keywords:** Convergence, Diversity, Many-objective optimization, Metaheuristic algorithm, Information feedback mechanism, Marine predator algorithm, Engineering, Mathematics and computing

## Abstract

Balancing diversity and convergence among solutions in many-objective optimization is challenging, particularly in high-dimensional spaces with conflicting objectives. This paper presents the Many-Objective Marine Predator Algorithm (MaOMPA), an adaptation of the Marine Predators Algorithm (MPA) specifically enhanced for many-objective optimization tasks. MaOMPA integrates an elitist, non-dominated sorting and crowding distance mechanism to maintain a well-distributed set of solutions on the Pareto front. MaOMPA improves upon traditional metaheuristic methods by achieving a robust balance between exploration and exploitation using the predator–prey interaction model. The algorithm underwent evaluation on various benchmarks together with complex real-world engineering problems where it showed superior outcomes when compared against state-of-the-art generational distance and hypervolume and coverage metrics. Engineers and researchers can use MaOMPA as an effective reliable tool to address complex optimization scenarios in engineering design. The MaOMPA source code is available at https://github.com/kanak02/MaOMPA.

## Introduction

The field of multi-objective optimization (MOO) maintains essential importance in engineering because systems demand optimally performed through balancing multiple competing objectives according to research by^1^. Evolutionary and swarm intelligence algorithms have emerged because of a need to handle complexity of MOO and these approaches have shown considerable effectiveness in this domain. These algorithms generate diverse high-quality solutions through their ability to achieve convergence while approaching the optimal front while maintaining solution diversity across this front. The need for algorithms to handle multiple objectives has grown substantially during recent years because complex optimization applications have entered fields like mechanical^2^ and structural engineering^3^, electronics and artificial intelligence.

The main difficulty in MOO occurs when managing objectives with numerous dimensions particularly when more than three objectives needs simultaneous evaluation. The performance of conventional algorithms deteriorates when standard selection pressures decrease because of these situations. Moreover, with many objectives, most solutions tend to be non-dominated, making it challenging to differentiate between them based solely on objective values. This issue leads to subpar results in convergence and diversity, which are essential for producing a robust set of solutions that comprehensively cover the Pareto front (POF)^4^.

One of the traditional approaches to MOO involves Pareto ranking, where each solution is evaluated based on whether others dominate it. The goal is to rank solutions in a way that favors non-dominated solutions (those that are not outperformed across all objectives) and select these solutions to build a diverse Pareto-optimal set. However, as the number of objectives increases, most solutions become non-dominated, reducing the effectiveness of Pareto ranking^5^. Consequently, diversity preservation mechanisms, such as crowding distance, have been introduced in algorithms like NSGA-II to address this issue^6^. Although this approach improves solution spread, its performance is limited in high-dimensional spaces^7^.

Another approach commonly used is decomposition-based strategies, where a multi-objective problem is broken down into numerous scalar optimization tasks, each targeting a single or a subset of objectives. This method, seen in algorithms like MOEA/D^8^, handles complex many-objective (MaO) problems by concurrently addressing simpler sub-tasks^9^. Decomposition-based strategies help improve solution diversity and distribution but also encounter limitations in adequately representing diverse solutions along the Pareto front. Generating weight vectors and choosing practical combination functions, such as weighted sum (WS) and Tchebycheff methods, add complexity and computational costs to these algorithms, mainly when objectives grow in number and diversity demands increase^10^.

To overcome these challenges, indicator-based algorithms like HypE use specific indicators (e.g., hypervolume) to balance convergence and diversity^11^. Hypervolume indicators are effective in MaO, as they measure the volume of the objective space dominated by the obtained solutions. However, hypervolume calculations are computationally intensive, growing exponentially with the number of objectives. Alternative indicators, like unary epsilon, offer different trade-offs, yet they may not always ensure the required diversity across high-dimensional Pareto fronts^12^.

In recent years, reference-based algorithms have emerged as another promising approach to addressing diversity^13^. Algorithms like NSGA-III use reference points and niche preservation to select solutions. In NSGA-III, reference points help maintain a well-distributed set of solutions by associating each solution with a specific point, ensuring solutions are selected from underrepresented areas of the search space^14^. Despite their advantages, these methods still face computational challenges when scaling to higher-dimensional problems and managing reference points can add complexity to the optimization process^15^.

Many-objective evolutionary algorithms (MaOEAs) are categorized into four main approaches: Pareto dominance-based, indicator-based, decomposition-based and reference vector-guided methods^16^. Pareto dominance-based algorithms, such as NSGA-II and SPEA-II, prioritize solutions based on dominance relationships, favoring non-dominated solutions. However, as the number of objectives increases, these methods face challenges in effectively distinguishing solutions, often requiring modifications like ε-dominance and grid-based approaches to maintain selection pressure and population diversity. Multi-indicator algorithms like SRA emerged because of this limitation to establish balanced indicator measurements that create better convergence with diverse results. The reference vector-based algorithms NSGA-III and RVEA implement dynamic reference vector systems that enhance the search process across diverse Pareto fronts and achieve uniform distribution of solutions. The MaOGBO method together with MOEA/D use adaptive mechanisms that modify weights or reference vectors while working with objective functions^17^. Researchers have developed single-objective metaheuristics specifically for MaO problems through the creation of the Red Deer Algorithm and Tree Growth Algorithm^18^. Studies indicate that MaOEA/D-ADA^20^ together with RVEA-DPS^21^ establish two algorithms which aim to balance convergence-diversity performance. The optimization algorithms achieve higher performance through adaptive procedures linked to advanced decomposition algorithms during complex optimization workloads. Reinforcement learning techniques enable the Multi-Objective Evolutionary Algorithm based on Reinforcement Learning (MOEA-RL)^22^ to achieve improved results for benchmark and real-world problems through adaptive search guidance. HMOA^23^ unites decomposition-based and indicator-based approaches into one algorithm^24^ structure which delivers scalable robust solutions for complex high-dimensional problems^25^.

Despite advancements in MaOEAs, challenges persist in balancing convergence and diversity, particularly in high-dimensional spaces. Some recent methods have introduced dynamic adaptation, chaos theory, or problem-specific criteria, aiming to enhance MaOEAs’ applicability to real-world, evolving optimization landscapes^19^.

The Marine Predators Algorithm (MPA),^26^ recently introduced for single-objective optimization, presents a novel approach to exploration and exploitation by modeling predator–prey interactions observed in marine ecosystems. While MPA has proven effective for single-objective problems, it lacks the mechanisms to handle multi-objective challenges, such as maintaining diversity across many dimensions. Nevertheless, the MPA framework offers valuable principles that can be adapted to create a new multi-objective approach^27^.

MPA serves as the base for Many-Objective Marine Predator Algorithm (MaOMPA) due to its unique characteristics that make it well-suited for many-objective optimization tasks. MPA draws its inspiration from marine predators’ natural foraging patterns that require them to balance their search between exploration and exploitation of prey resources. The natural foraging model within MPA allows the algorithm to explore complex search areas effectively. MPA divides its operation into three distinct phases of high-velocity exploration followed by balanced exploration–exploitation then low-velocity exploitation and these phases adapt automatically according to the optimization process. The staged operation of MPA helps prevent premature convergence while preserving diversity because many-objective optimization requires effective convergence and diversity management. MPA implements fish aggregating device effect mechanisms that create sudden search space jumps to stop problems caused by local optima traps. The feature enables the algorithm to explore multiple regions of the search space effectively so it works well with complex optimization problems of high dimensionality. MPA uses predator–prey dynamics as a natural means to manage exploration and exploitation activities to produce stable many-objective optimization outcomes. The predator–prey mechanisms throughout MPA separate this algorithm from PSO and GA since these methods generally fail to sustain proper exploration–exploitation equilibrium across multiple dimensions. MaOMPA demonstrates how MPA became applicable to many-objective optimization through its implementation of strengths which specifically address many-objective problem requirements. MaOMPA uses elitist non-dominated sorting together with crowding distance mechanisms to generate a well-distributed set of solutions that appear on the Pareto front. The algorithm uses adaptive predator–prey dynamics to boost exploration at the beginning then transitions toward exploitation while the algorithm approaches convergence. The modified version of MaOMPA shows exceptional performance when dealing with complex many-objective problems because its enhancements enable it to maintain solution diversity and convergence.

In this paper, MaOMPA was tested across standard benchmark functions and real-world engineering problems, such as WFG1 to WFG9 benchmark test functions^28^ and four real world RWMAOP1 to RWMAOP4 problems.^29^ These benchmarks provide a robust assessment of MaOMPA’s effectiveness in addressing constrained and unconstrained optimization scenarios. The performance of MaOMPA was compared against state-of-the-art algorithms, like MaOALO (Many-Objective Ant-Lion Optimization Algorithm)^30^ and MaOPSO (Many-Objective Particle Swarm Optimization)^31^ and MaOSOS (Many-Objective Symbiotic organism search Optimization)^32^ and MaOMVO (Many-Objective Multi-Verse Optimizer)^33^ and NSGA-III (Non-dominated Sorting Genetic Algorithm III)^14^.

focusing on metrics such as Generational Distance^34^, spread^35^, hypervolume^36^, Inverted Generational Distance^37^ and runtime. In these tests, MaOMPA demonstrated superior convergence rates, hypervolume metrics and improved solution diversity on the Pareto front, highlighting its competitive performance and practical applicability.

The key contributions of this study are as follows:

- This study introduces MaOMPA as a many-objective adaptation of the Marine Predators Algorithm, enhancing it for MaO by incorporating non-dominated sorting, crowding distance and predator–prey dynamics.
- MaOMPA’s adaptive phases, inspired by predator–prey interactions, enhance its ability to explore diverse regions of the search space before shifting to focused exploitation, thereby maintaining a balanced and comprehensive Pareto front.
- Rigorous benchmark function and engineering problem tests enabled MaOMPA to prove its superiority by generating better results than traditional algorithms in terms of generational distance, hypervolume and diversity metrics.
- MaOMPA proves itself as both robust and efficient for high-dimensional optimization tasks in engineering applications which provides practitioners and researchers in mechanical design resource management and environmental modeling with a valuable tool.

## Marine predators algorithm

**Fig. 1:**
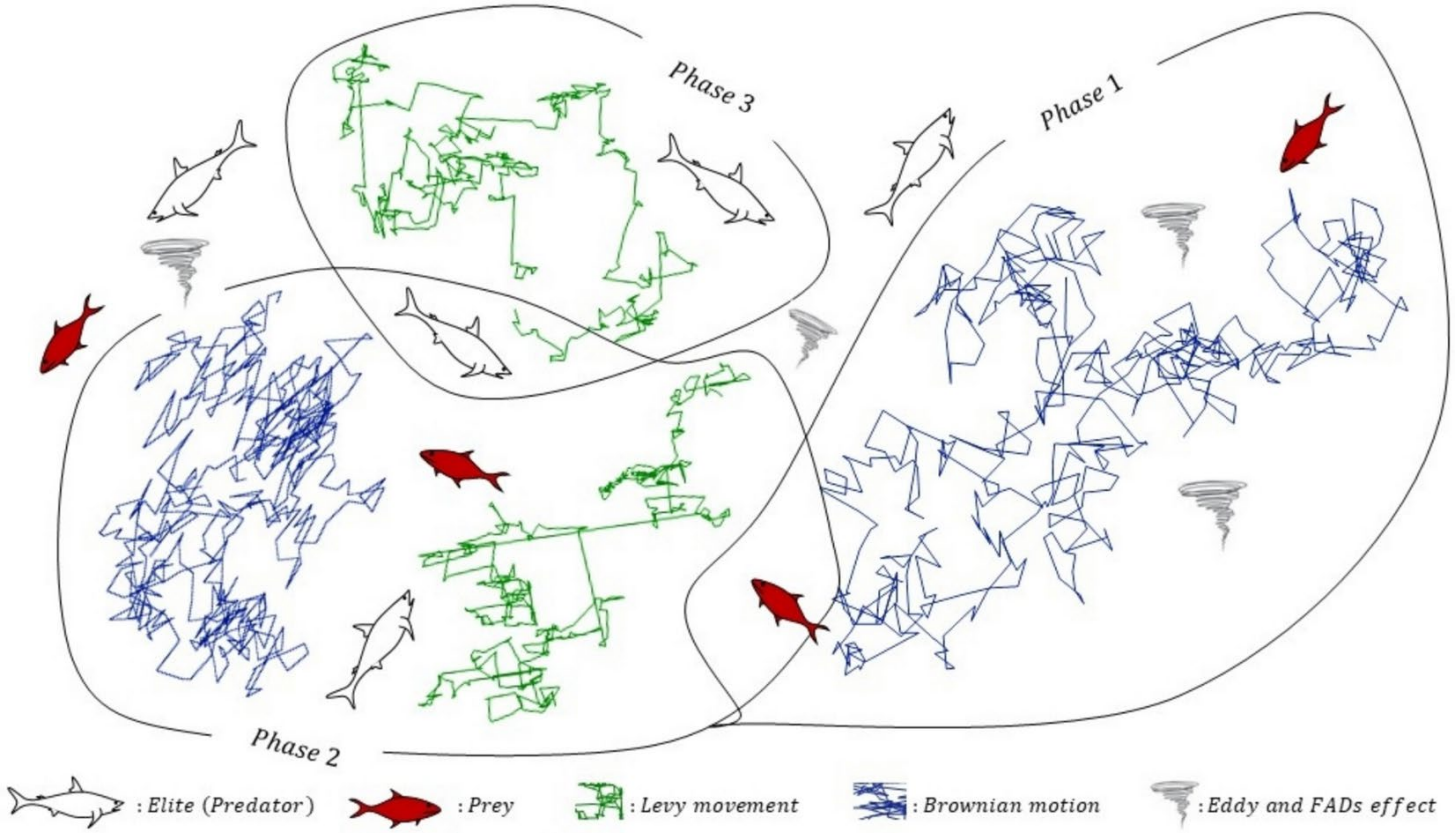
Graphical overview of different phases of MPA.

MPA functions as a metaheuristic optimization method which derives its principles from how marine predators hunt in nature. This algorithm applies predator–prey natural observations to maintain the balance of exploration and exploitation which leads to optimal widespread solutions across multiple optimization objectives. The following section demonstrates the mathematical description of the MPA algorithm. Figure 1 shows the MPA’s three phases that employ Brownian motion initially followed by Brownian and Lévy movement combination in Phase 2 preceding focused Lévy movement in Phase 3 for modeling predator search behavior affected by environment-induced eddy currents and aggregation devices.

### Initialization of search agents (predators and prey)

The algorithm begins by initializing a population of search agents (marine predators). Each agent represents a potential solution in the search space.

- Define the population size $N$, dimensionality $D$ and search space bounds $[{X}_{\text{min}},{X}_{\text{max}}]$.
- Randomly initialize each search agent’s position ${X}_{i}$ within the search space as per Eq. 1.
  1 $${X}_{i}={X}_{\text{min}}+{\text{rand}}(\text{0,1})\times ({X}_{\text{max}}-{X}_{\text{min}})$$

where ${\text{rand}}(\text{0,1})$ generates random values between 0 and 1.

### Fitness evaluation and selection of elite agent

Evaluate the fitness of each agent according to the objective function $f(X)$ and identify the best solution (elite) in the initial population. This elite agent denoted as ${X}_{\text{elite}}$, represents the “top predator” in the current iteration.

### Marine predator dynamics: three-stage movement

The MPA algorithm simulates three distinct phases in marine predator behavior. These phases correspond to different movement strategies that balance exploration (searching new areas) and exploitation (intensifying search in promising regions).

### Phase 1: high-velocity ratio (exploration phase)

In the initial stage, the predators move faster than the prey, leading to a high-velocity exploration phase. This is mathematically modeled as per Eq. 2.

1. Calculate step size:

2 $${\text{StepSize}}_{i}={\text{RB}}\otimes ({X}_{\text{elite}}-{\text{RB}}\otimes {X}_{i})$$

where: ${\text{RB}}$ is a vector of random numbers from a normal distribution representing Brownian motion. $\otimes$ denotes element-wise multiplication.

2. Update position:

3 $${X}_{i}={X}_{i}+P\times {\text{rand}}(\text{0,1})\otimes {\text{StepSize}}_{i}$$

where $P$ is a constant that scales the movement, typically set to 0.5.

This phase occurs in the first third of the iterations shown by Eq. 3, promoting exploration by encouraging large, random movements.

### Phase 2: unit velocity ratio (transition from exploration to exploitation)

In the middle stage of the algorithm, the predators and prey move at approximately the same velocity, indicating a balance between exploration and exploitation. The population is divided into two halves, each adopting different movement strategies.

1. For the first half of the population (Lévy movements):

4 $${\text{StepSize}}_{i}={\text{RL}}\otimes ({X}_{\text{elite}}-{\text{RL}}\otimes {X}_{i})$$

where: ${\text{RL}}$ represents Lévy distribution-based random numbers in Eq. 4.

2. Update position as per Eq. 5.

5 $${X}_{i}={X}_{i}+P\times {\text{rand}}(\text{0,1})\otimes {\text{StepSize}}_{i}$$

3. For the second half of the population (Brownian movements):

6 $${\text{StepSize}}_{i}={\text{RB}}\otimes ({X}_{\text{elite}}-{X}_{i})$$

Update position:

7 $${X}_{i}={X}_{i}+P\times {\text{CF}}\otimes {\text{StepSize}}_{i}$$

where ${\text{CF}}$ is an adaptive control factor for step size in Eq. 7.

This phase occurs in the middle third of the iterations, allowing exploration and exploitation based on the nature of the search space as per Eq. 6.

### Phase 3: low-velocity ratio (exploitation phase)

In the final stage, the predators move slower than the prey, focusing on exploitation. This phase is designed to refine solutions by concentrating on regions close to the elite predator, avoiding premature convergence to local optima.

1. Step size:

8 $${\text{StepSize}}_{i}={\text{RL}}\otimes ({X}_{\text{elite}}-{X}_{i})$$

2. Position update:

9 $${X}_{i}={X}_{\text{elite}}+P\times {\text{CF}}\otimes {\text{StepSize}}_{i}$$

This stage refines the population by leveraging smaller, controlled movements toward the elite agent, enhancing exploitation as demonstrated in Eq. 8 and 9.

### Environmental influence (fish aggregating device, FAD effect)

The algorithm incorporates a behavioral change called the FAD effect to prevent the search agents from stagnating in local optima. This mechanism enables the agents to perform a sudden, significant jump to explore new regions.

10 $${X}_{i}=\left\{\begin{array}{ll}{X}_{i}+{\text{CF}}\times \left({X}_{\text{min}}+{\text{rand}}(\text{0,1})\otimes ({X}_{\text{max}}-{X}_{\text{min}})\right),& \text{if }r<{\text{FADs}}\\ {X}_{i}+(FADs\times (1-r)+r)\times ({X}_{r1}-{X}_{r2}),& {\text{otherwise}}\end{array}\right.$$

where: $FADs$ is the probability of the FAD effect, typically set around 0.2. $r$ is a random value in $[\text{0,1}]$. ${X}_{r1}$ and ${X}_{r2}$ are randomly selected search agents in the population in Eq. 10.

This effect simulates marine predators’ ability to revisit previously successful areas, thus improving the diversity of the search agents.

### Iteration and termination and output

The algorithm performs this sequence of phases for either a specified number of iterations or until the objective function values achieve the established convergence criteria. The algorithm produces the elite agent’s position together with its fitness value which represents the optimal solution discovered by MPA.

The step-by-step mathematical MPA model represents its fundamental operational principles through a process which duplicates natural marine predator movement dynamics between exploration and exploitation phases. The algorithm’s design enables efficient search of complex optimization landscapes which makes it applicable to multiple optimization problems.

### Proposed many-objective marin predators algorithm (MaOMPA)

The MaOMPA algorithm initiates by generating an initial population of $N$ random solutions, aiming to optimize across $M$ objectives. The algorithm also involves $p$ partitions and utilizes a set of reference points derived using the Das and Dennis method, with the formula $H=\frac{(M+p-1)!}{p!(M-1)!}$, ensuring that $H\approx N$ to align with the population size. Let ${x}_{t,i}^{k}$ represent the $i$-th individual of the $k$-th subpopulation in the current generation $t$ and ${x}_{t+1,i}^{k}$ denote the same individual at the next generation $t+1$, which is generated through the MPA process.

The fitness of the $(t+1)$-th generation individual ${x}_{t+1,i}^{k}$ is represented as ${f}_{t+1,i}$ and the set ${U}_{t+1}$ comprises all individuals in this generation. Using the Multi-Population Algorithm (MPA) operations and an Intermediate Fusion Mechanism (IFM), we can compute the updated position ${x}_{t+1,i}^{k}$ based on the current and neighboring populations, as follows:

$${x}_{t+1,i}^{k}={\alpha }_{1}{x}_{t,i}^{k}+{\alpha }_{2}{x}_{t,j}^{k}, {\text{where}} {\alpha }_{1}+{\alpha }_{2}=1$$

with weight coefficients calculated as per Eq. 11.

11 $${\alpha }_{1}=\frac{{f}_{t,i}^{k}}{{f}_{t,i}^{k}+{f}_{t,j}^{k}}, {\alpha }_{2}=\frac{{f}_{t,j}^{k}}{{f}_{t,i}^{k}+{f}_{t,j}^{k}}$$

In this formulation, ${x}_{t}^{k}$ represents the $k$-th individual selected from generation $t$, while ${f}_{t}^{k}$ is the fitness value of that individual. The parameters ${\alpha }_{1}$ and ${\alpha }_{2}$ are weight coefficients that guide the offspring’s generation by influencing selected parents’ contributions. This creates an offspring population ${Q}_{t+1}$ and the union of current and offspring populations is denoted by ${R}_{t}={P}_{t}\cup {Q}_{t}$.

The combined population ${R}_{t}$ is then sorted into $w$-non-dominated levels, represented as ${F}_{1},{F}_{2},...,{F}_{w}$. Starting with the first front ${F}_{1}$, all individuals in levels up to $l$ are added to a new set ${S}_{t}$, while the remaining individuals in ${R}_{t}$ are discarded. If $|{S}_{t}|=N$, meaning the size of ${S}_{t}$ matches the initial population size, no further actions are necessary and the next generation begins with ${P}_{t+1}={S}_{t}$. Otherwise, additional individuals from the next fronts are included in ${P}_{t+1}={S}_{t}\cup {F}_{l}$ and any remaining $(K=N-|{P}_{t+1}|)$ individuals are selected from the last front ${F}_{l}$ using a niche-preserving operator.

Each member of the new population ${P}_{t+1}$ and the selected individuals from front ${F}_{l}$ are then normalized according to the current distribution of objective values, as specified in Algorithm 2. This normalization step ensures uniformity in objective vectors and reference points across the population, promoting well-distributed solutions. Subsequently, each individual is mapped to a specific reference point by calculating the shortest perpendicular distance (d()), as described in Algorithm 3, thus establishing a reference line between each solution and the reference points.

To enhance diversity, a strategic niching approach (outlined in Algorithm 5) is applied to select individuals in ${F}_{l}$ that are linked to under-represented reference points. This selection is based on a niche count ${\rho }_{i}$ calculated for each reference point in ${P}_{t+1}$. If the termination criteria are not met, the process repeats, generating a new parent population ${P}_{t+1}$ to create the subsequent population ${Q}_{t+1}$. This selection approach adds computational complexity, scaling approximately as ${N}^{2}{\text{log}}^{2}(N)$ or $O({N}^{2}M)$, but it enhances the solution’s quality by maintaining diversity across objectives.

**Algorithm 1:**
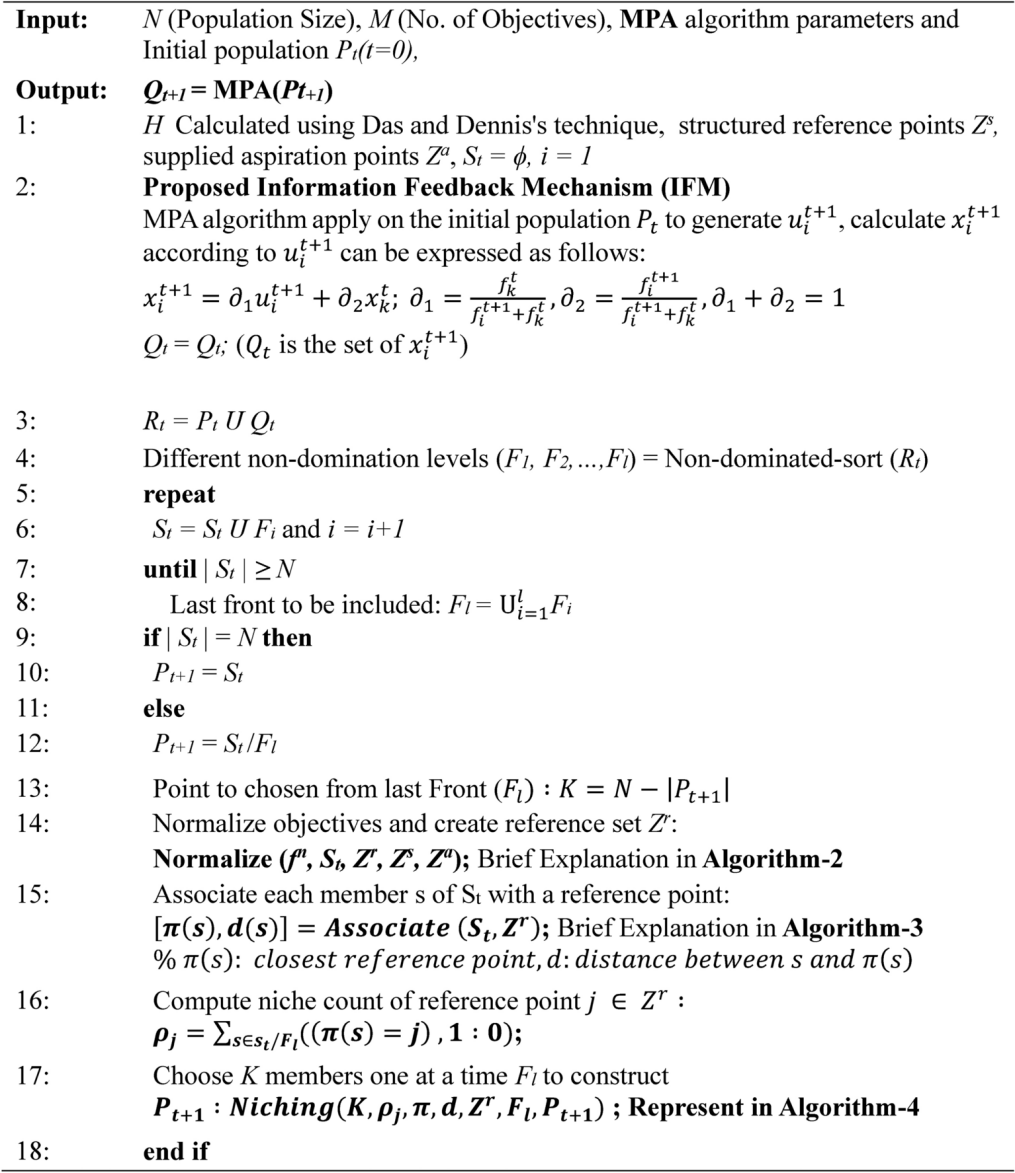
Generation t of MaOMPA algorithm with IFM procedure.

**Algorithm 2:**
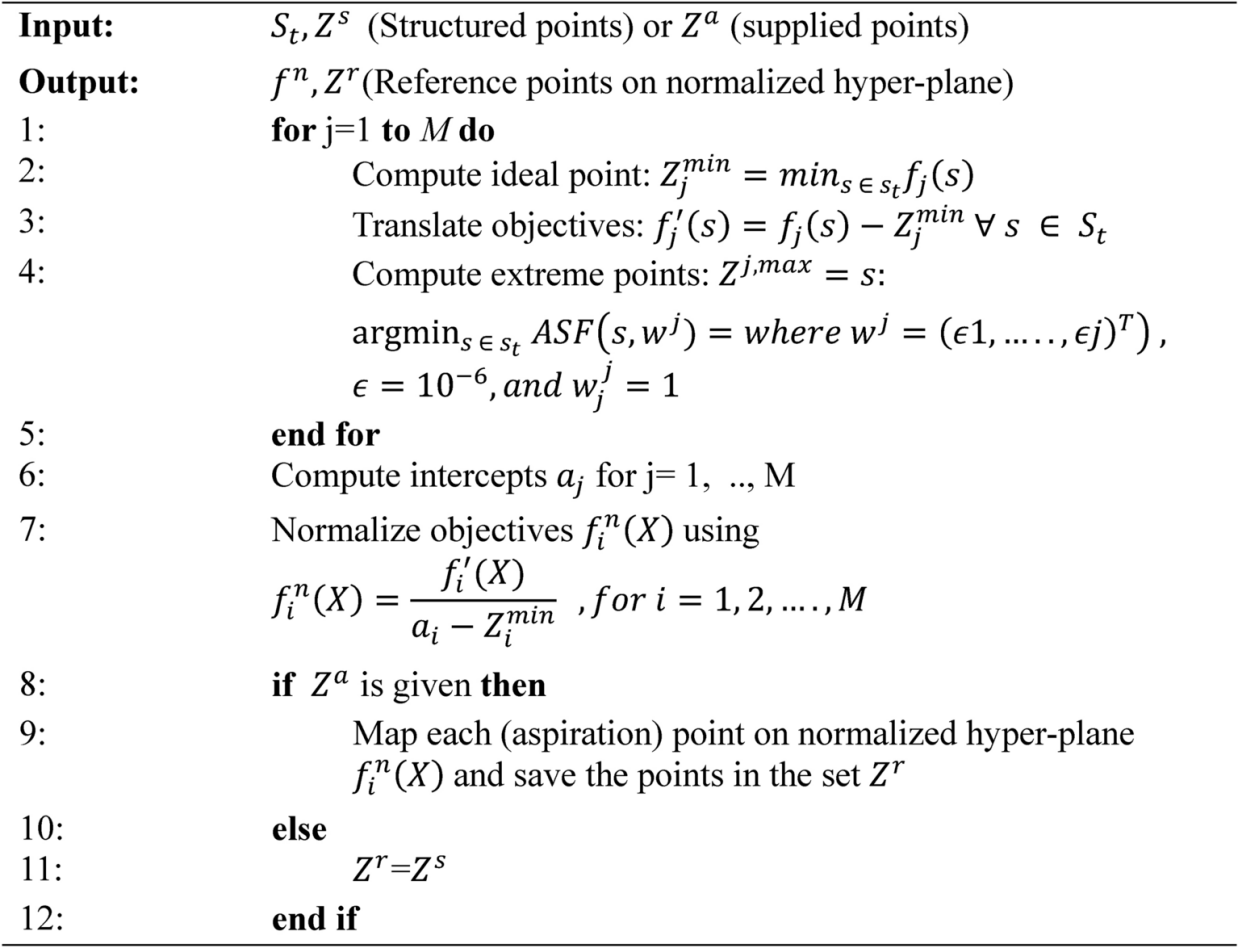
Normalize (*f*^*n*^,*S*_*t*_,*Z*^*r*^,*Z*^*s*^/*Z*^*a*^ ) procedure.

**Algorithm 3:**
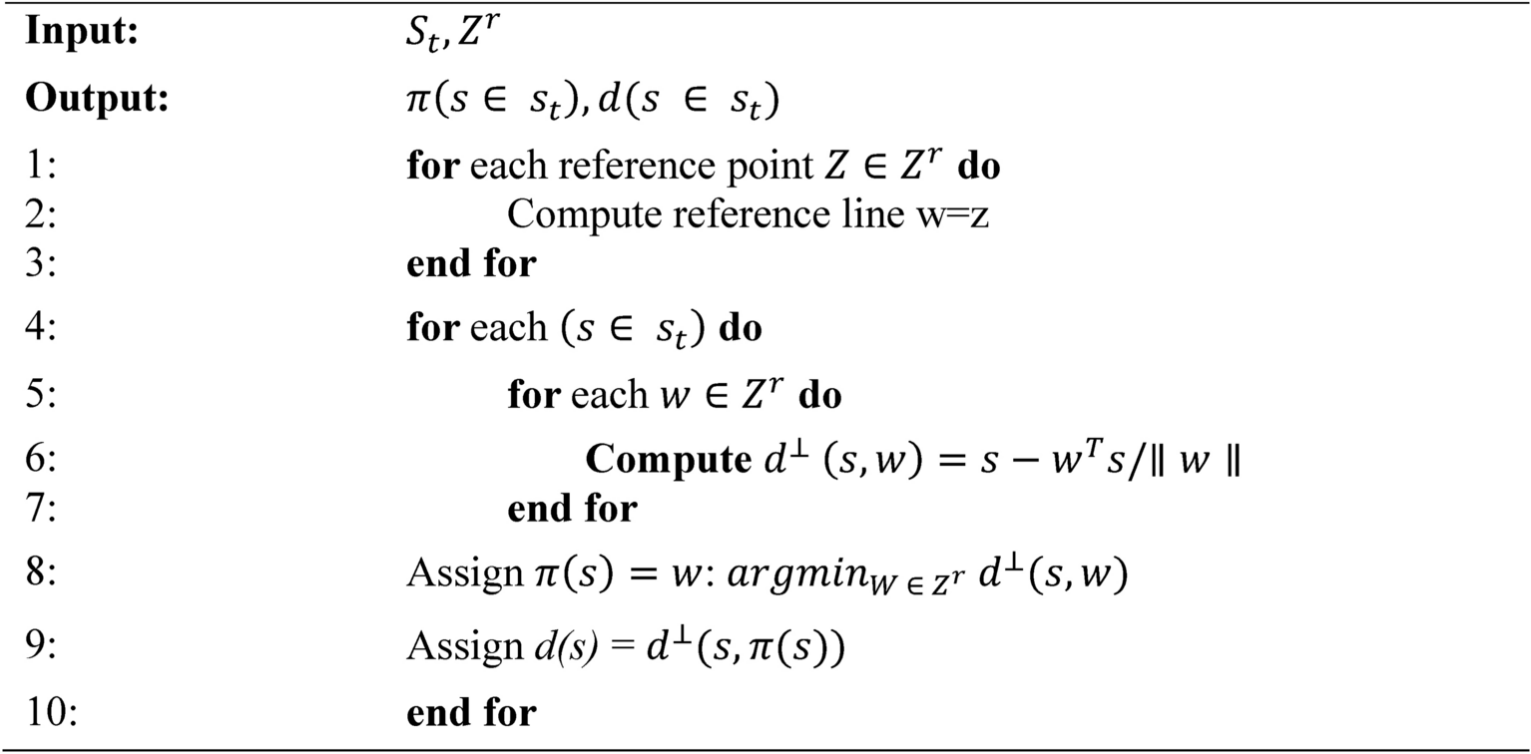
Associate (*S*_*t*_,*Z*^*r*^) procedure.

**Algorithm 4:**
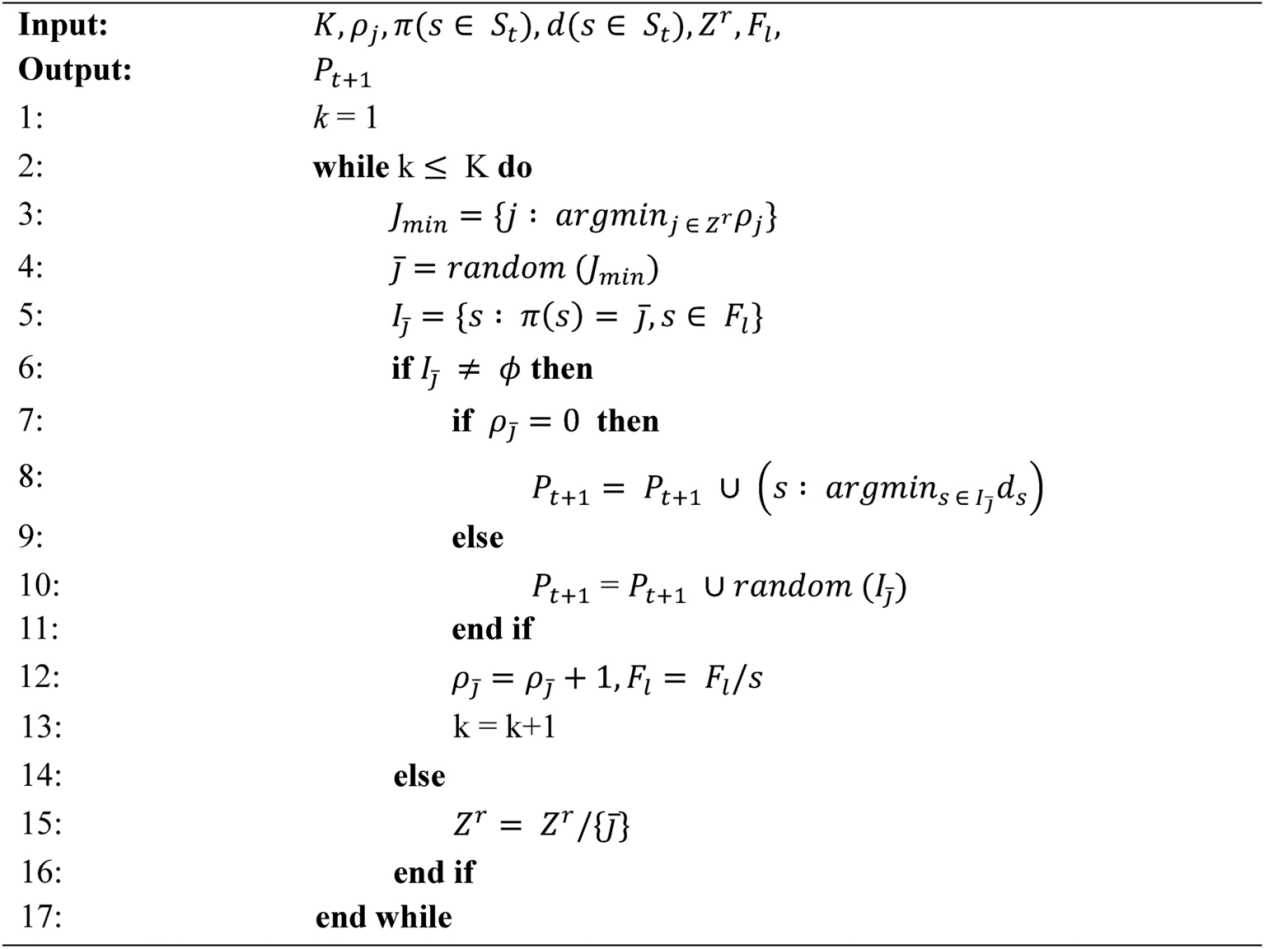
Niching (*K,ρ*_*j*_,*π,d,Z*^*r*^,*F*_*l*_,*P*_(*t+1*)_) procedure.

Algorithm 1 introduces the main generation process of MaOMPA, incorporating an Information Feedback Mechanism (IFM) that refines population updates by combining current and newly generated individuals through specific weighting based on objective values. This algorithm applies non-dominated sorting and crowding distance mechanisms to ensure selection diversity. Algorithm 2 focuses on normalizing the objective values, mapping them to a reference hyper-plane by calculating ideal and extreme points to facilitate comparison across objectives. Algorithm 3 associates each individual with reference points to determine their positional alignment in objective space, which aids in the selection of diverse solutions. Algorithm 4 handles the niching process, which balances exploration by selecting individuals based on their crowding distance and proximity to reference points, enhancing solution spread across the Pareto front.

**Fig. 2:**
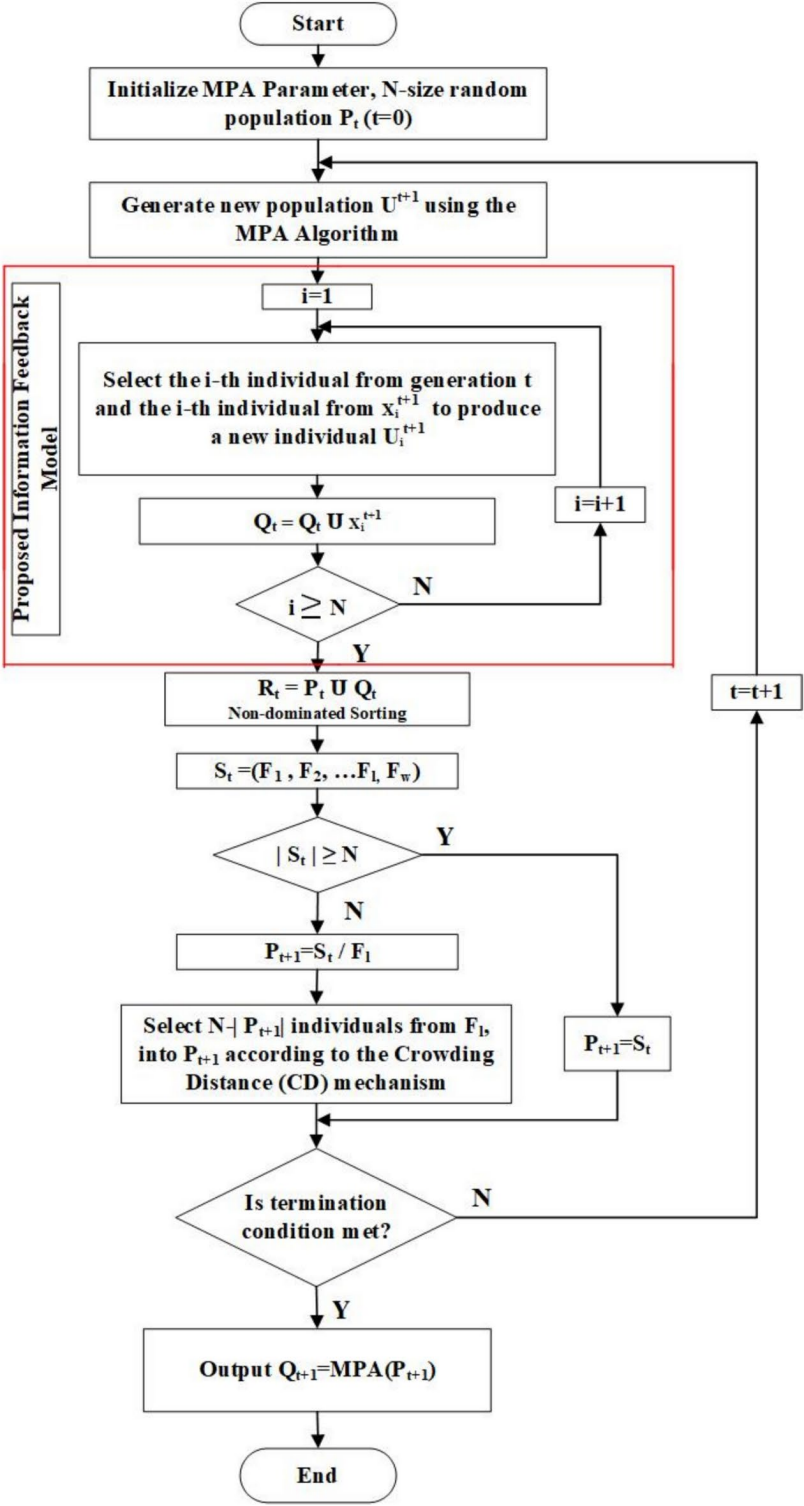
Flowchart of proposed MaOMPA.

The flowchart in Fig. 2 provides a step-by-step visualization of MaOMPA, starting from the initialization of MPA parameters and population. The algorithm iteratively generates a new population, applying IFM to update individuals and combines current and updated populations for non-dominated sorting. It evaluates the crowding distance and selects the next generation based on diversity and non-dominance, continuing until termination conditions are met. The output yields a diversified Pareto-optimal front, demonstrating MaOMPA’s capacity to handle complex, MaO landscapes. This flowchart serves as a comprehensive guide for implementing the proposed algorithm by outlining key procedural steps.

## Results and discussion

### Benchmark WFG problems

Both standard benchmark and actual engineering design problems were used to test the performance and stability of the MaOMPA. The evaluation uses WFG test suite which includes WFG1 through WFG9. Each WFG test suite MaF problem contains $(k +M-1)$ decision variables when M stands for the total number of objective functions. The design enables decision space size adjustments according to the number of objectives present. The WFG problems require decision variables to be defined through the combination of $(k+l)$ where $k$ equals $\left(M - 1\right)$ and $l$ has a value of 10. The system design guarantees that WFG test suite requirements are met by each problem configuration while demanding effective management of high-dimensional objective spaces from the algorithm.

In addition to the WFG benchmarks, four real-world many-objective engineering design problems are included to validate the MaOMPA’s performance in practical applications. These real-world problems consist of—

1. The Car Cab Design (RWMaOP1)^29^ problem requires optimization of automobile cab design to fulfill various performance and aesthetic requirements for developing efficient and ergonomic vehicles.

2. The 10-Bar Truss Structure (RWMaOP2)^38^ represents a structural optimization problem which combines weight reduction with stability enhancement through various design parameters for engineering application assessment.

3. Water and Oil Repellent Fabric Development (RWMaOP3)^39^ problem focuses on designing fabric with enhanced repellence properties, balancing material costs, durability and repellent effectiveness, which is crucial for advanced textile manufacturing.

4. Ultra-Wideband Antenna Design (RWMaOP4)^40^ is about the design of an ultra-wideband antenna is optimized to achieve specific performance criteria, such as frequency response and gain, which is essential in telecommunications and radar applications.

### Comparison algorithms and parameter settings

The MaOMPA performance evaluation takes place through experimental comparisons with different advanced many-objective optimization algorithms (MOAs). The evaluation uses MaOALO, MaOPSO, MaOSOS, MaOMVO and NSGA-III. The recognized performance of these competing algorithms in MaO problem-solving serves as an extensive benchmark to evaluate the capabilities of MaOMPA.

The testing environment consists of MATLAB R2020a operating on a system with Intel Core i7-9700 CPU. The testing environment provides uniform computational results for every experimental run. The algorithms run 30 independent times for every test case to achieve reliable statistical results. The method allows researchers to handle performance fluctuations that emerge from the stochastic behaviour of the algorithms. The selection of population size depends on M which represents the number of objective functions in each problem. The optimization problems with 5, 7 and 9 objectives require population sizes of N = 210, N = 156 and N = 276 respectively. The population size scaling mechanism ensures adequate search space diversity by adapting to the number of objectives in MaO problems. Each test instance has a maximum function evaluation limit set at 10,000. The maximum computational budget limitation standardizes algorithm performance because it provides equivalent operational resources for evaluating their efficiency and convergence ability throughout the evaluation period. The 10,000 MaxFEs cap strikes an equilibrium between efficient computations and detailed exploration to let analysts compare solution quality beyond excessive computing time. A thorough experimental method with strong comparative analysis evaluates MaOMPA’s performance to demonstrate its effectiveness when compared to prominent other MOAs for solving MaO problems of high dimensionality.

### Experimental results on WFG problems

**Fig. 3:**
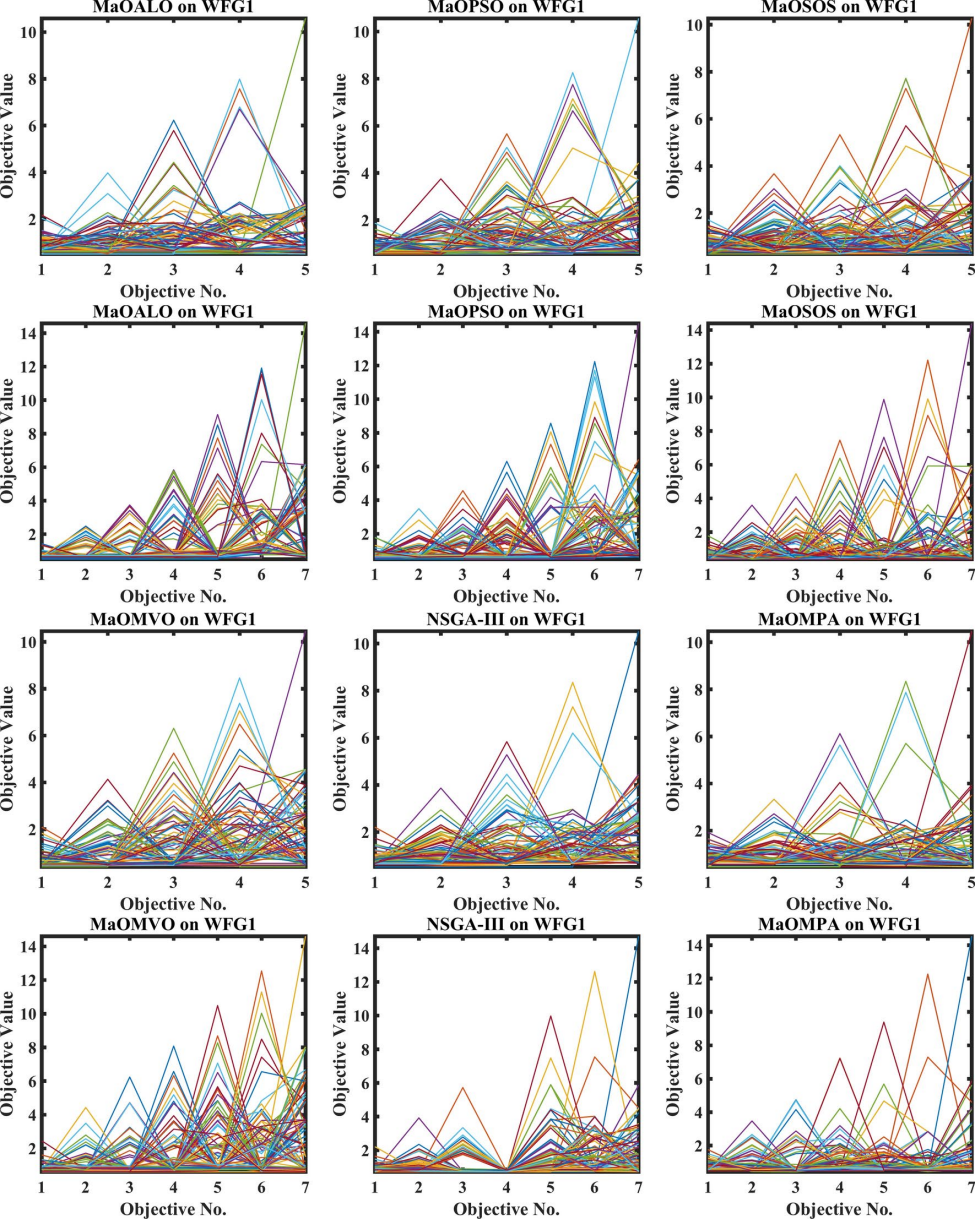

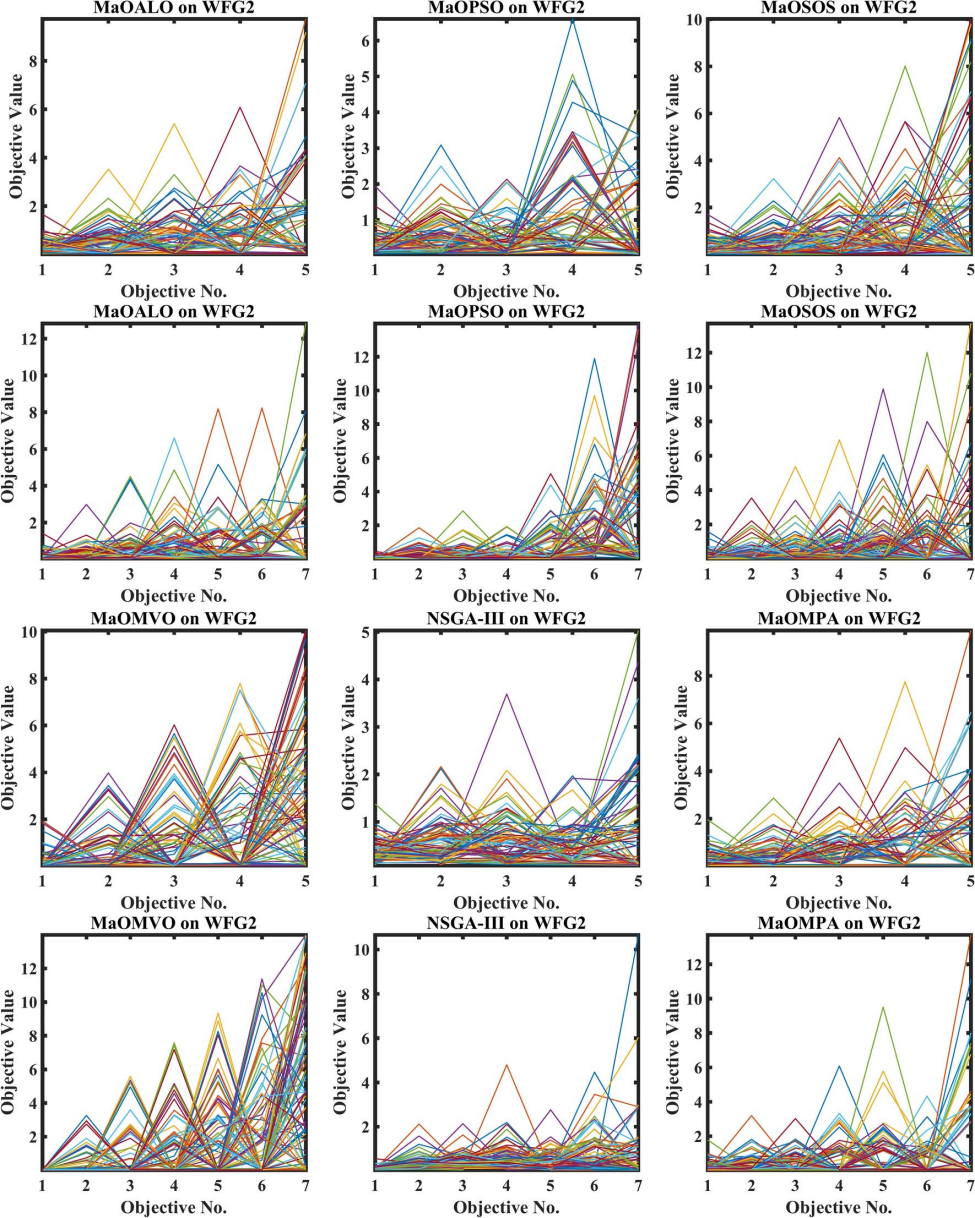

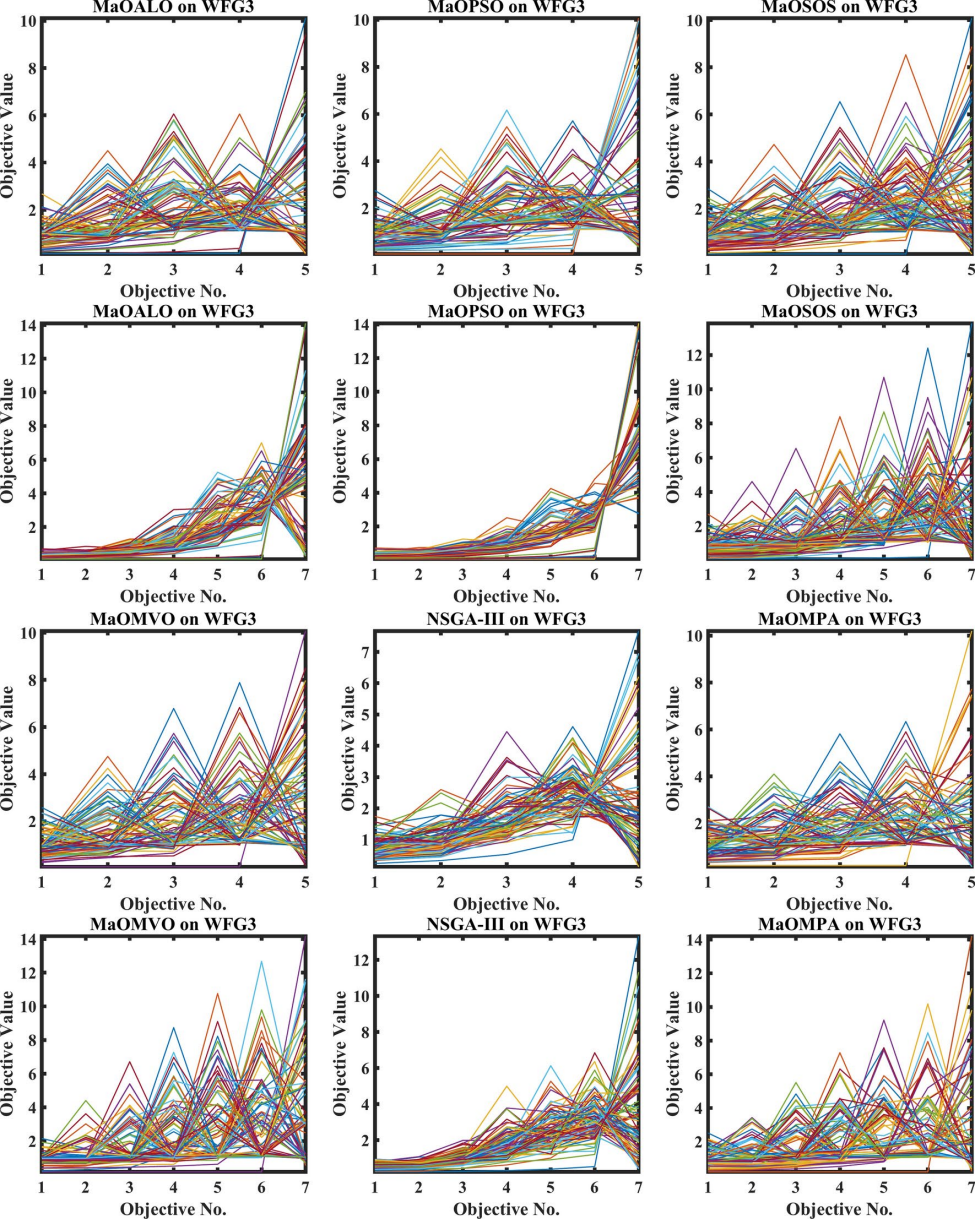

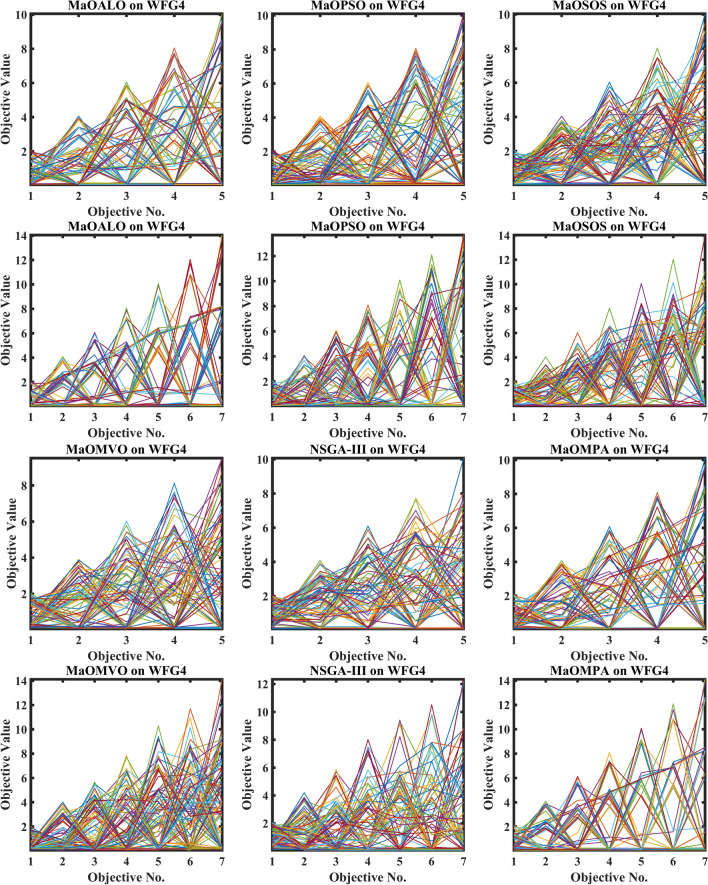

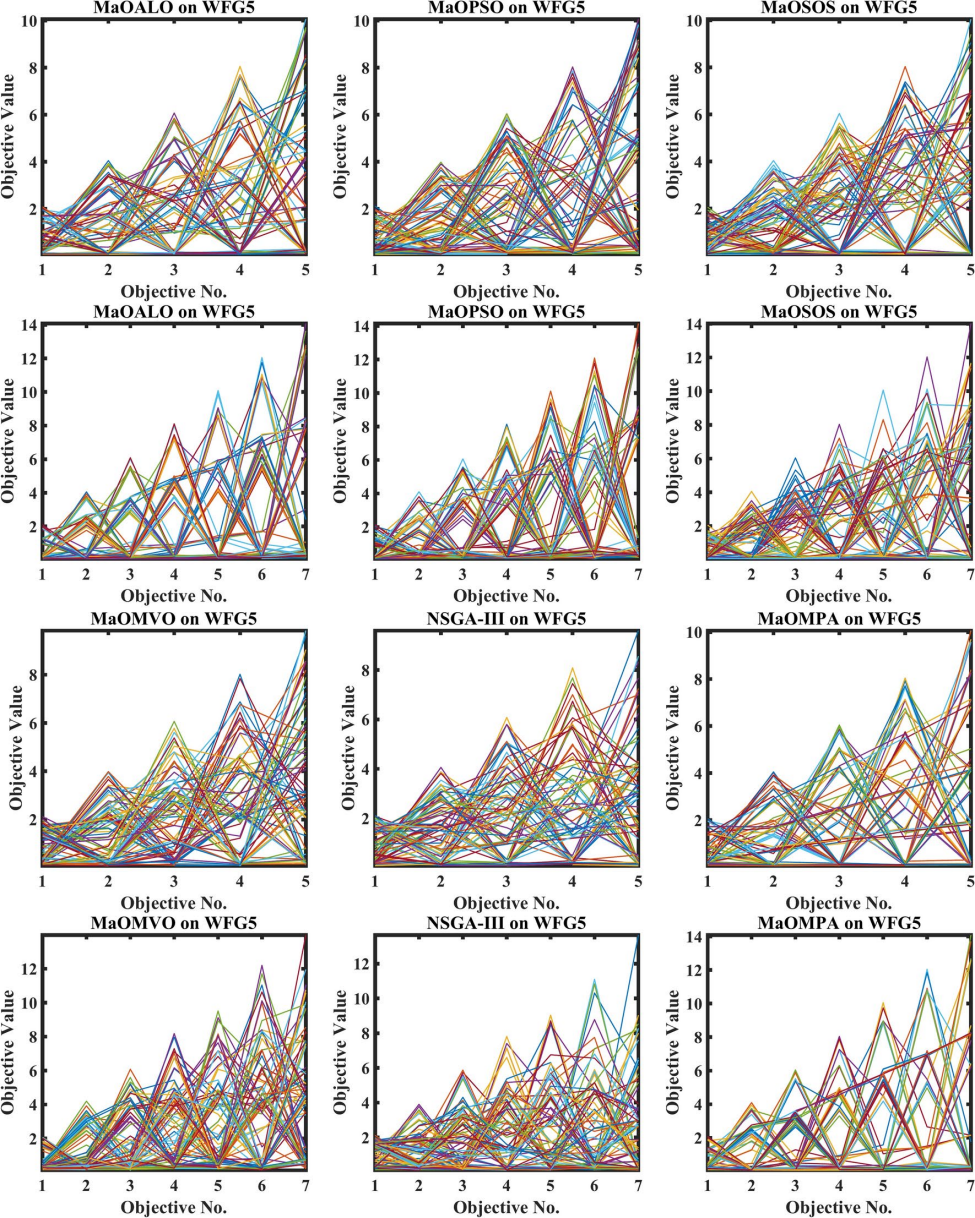

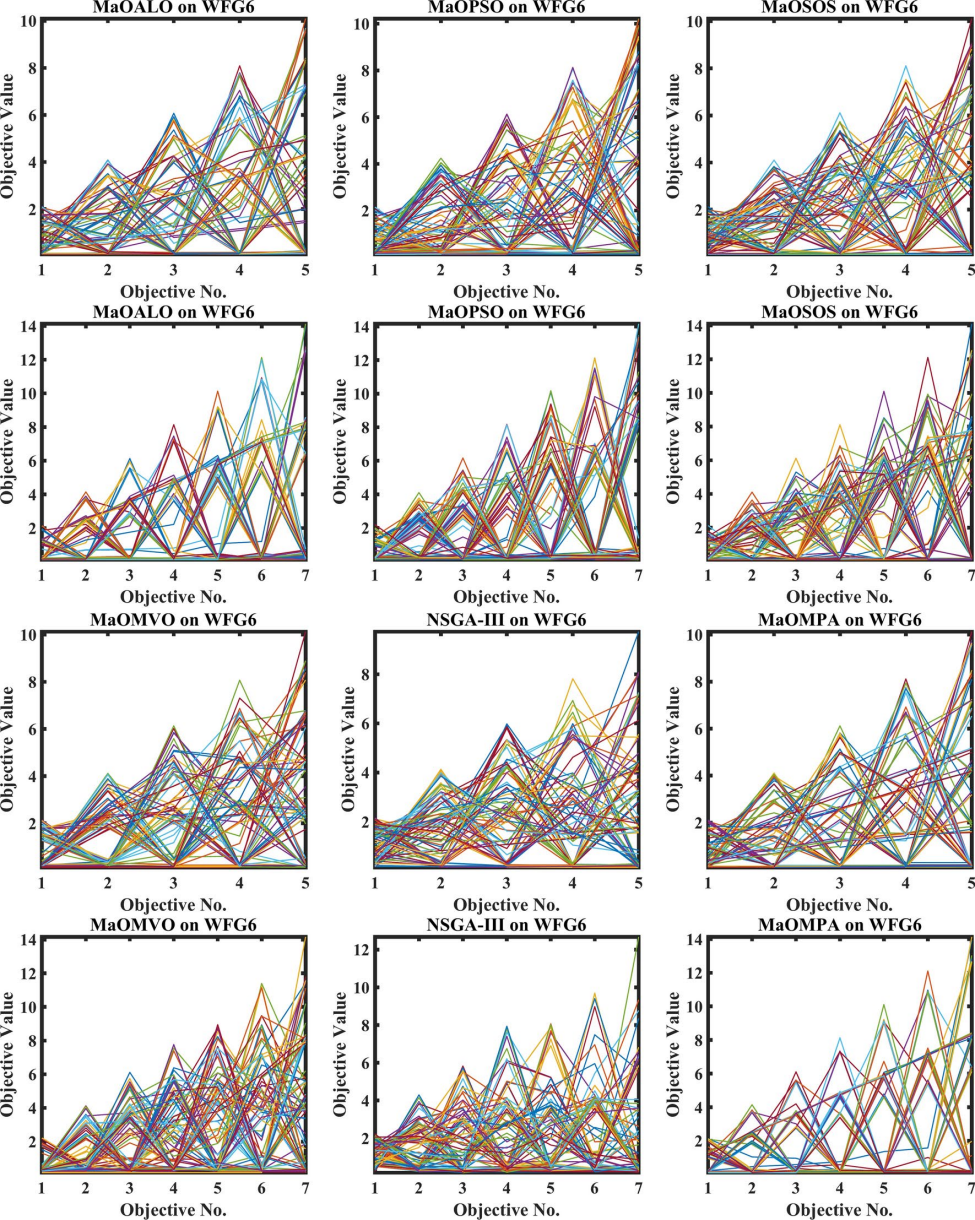

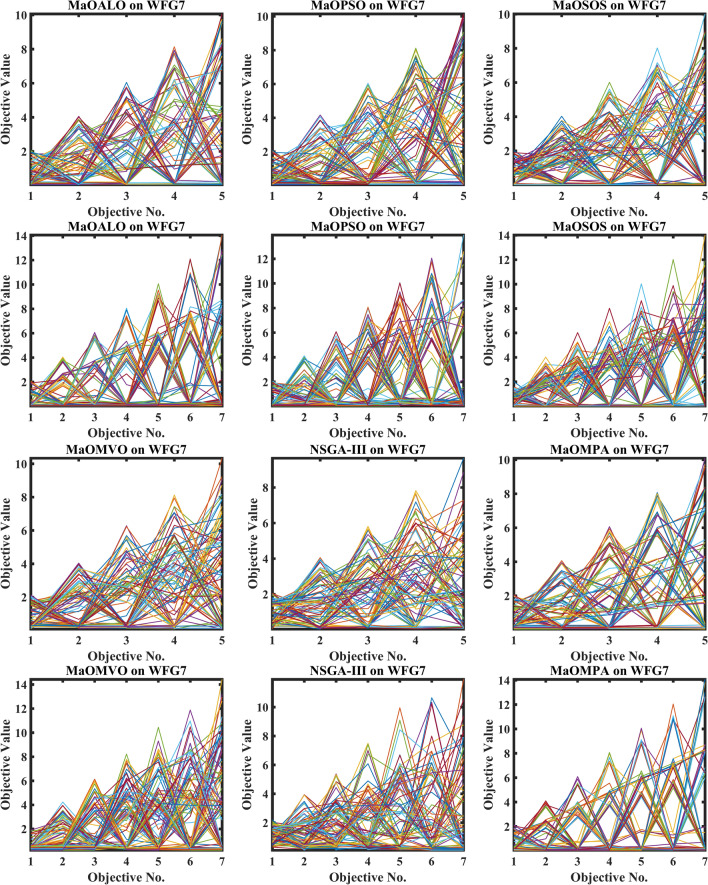

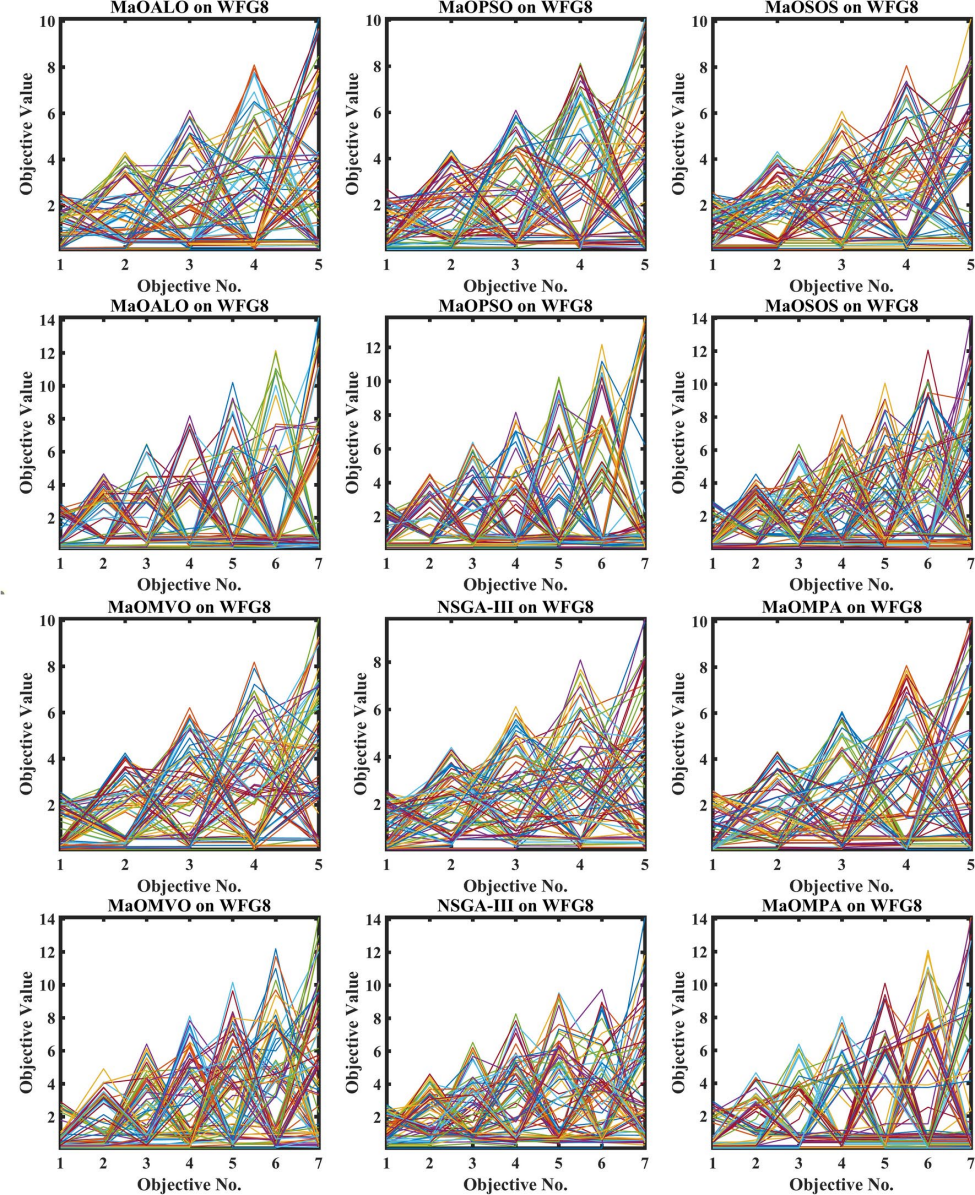

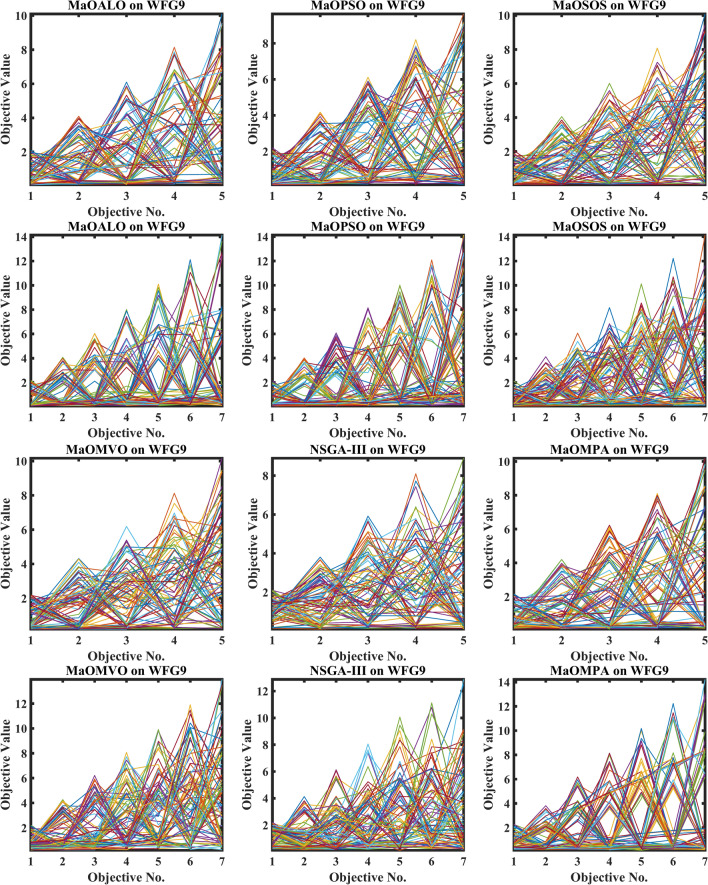
Best pareto optimal front obtained by different algorithms on MaF problems.

Figure 3 demonstrates how different algorithms achieve Pareto optimal fronts when solving MaF problems and WFG1 to WFG9 benchmarks through evaluation of convergence and objective diversity. The MaOMPA demonstrates an extensive solution spread which effectively covers the Pareto front better than NSGA-III, MaOPSO, MaOALO and MaOMVO across different objective numbers. The MaOMPA algorithm delivers solutions that precisely represent the true Pareto front throughout various high-dimensional spaces in all tests. MaOMPA demonstrates superior performance by maintaining diverse solutions while achieving optimal results in its distribution pattern. The balance achieved by MaO problems becomes essential for exploring different trade-offs when dealing with increasing numbers of objectives.

MaOMPA achieves superior performance in both solution diversity and convergence when used to solve WFG1 through WFG9 benchmark problems that have different objective counts when compared against other algorithms. The WFG functions present an extensive challenge to algorithms because they combine sophisticated structures with multiple Pareto front variations. The MaOMPA algorithm demonstrates better performance in reaching optimal Pareto fronts across WFG problems with 5, 7 and 9 objectives while handling distinctive landscape characteristics. The adaptive predator–prey dynamics of MaOMPA enable its search process to move from exploration to exploitation during algorithm progression thus minimizing premature convergence. MaOMPA’s use of crowding distance mechanisms enhances its ability to maintain a well-spread front across high-dimensional objective spaces, outperforming other algorithms in convergence and diversity metrics. These results, illustrated in Fig. 3, affirm MaOMPA’s effectiveness in achieving balanced solutions in synthetic and real-world MaO problems.

**Fig. 4:**
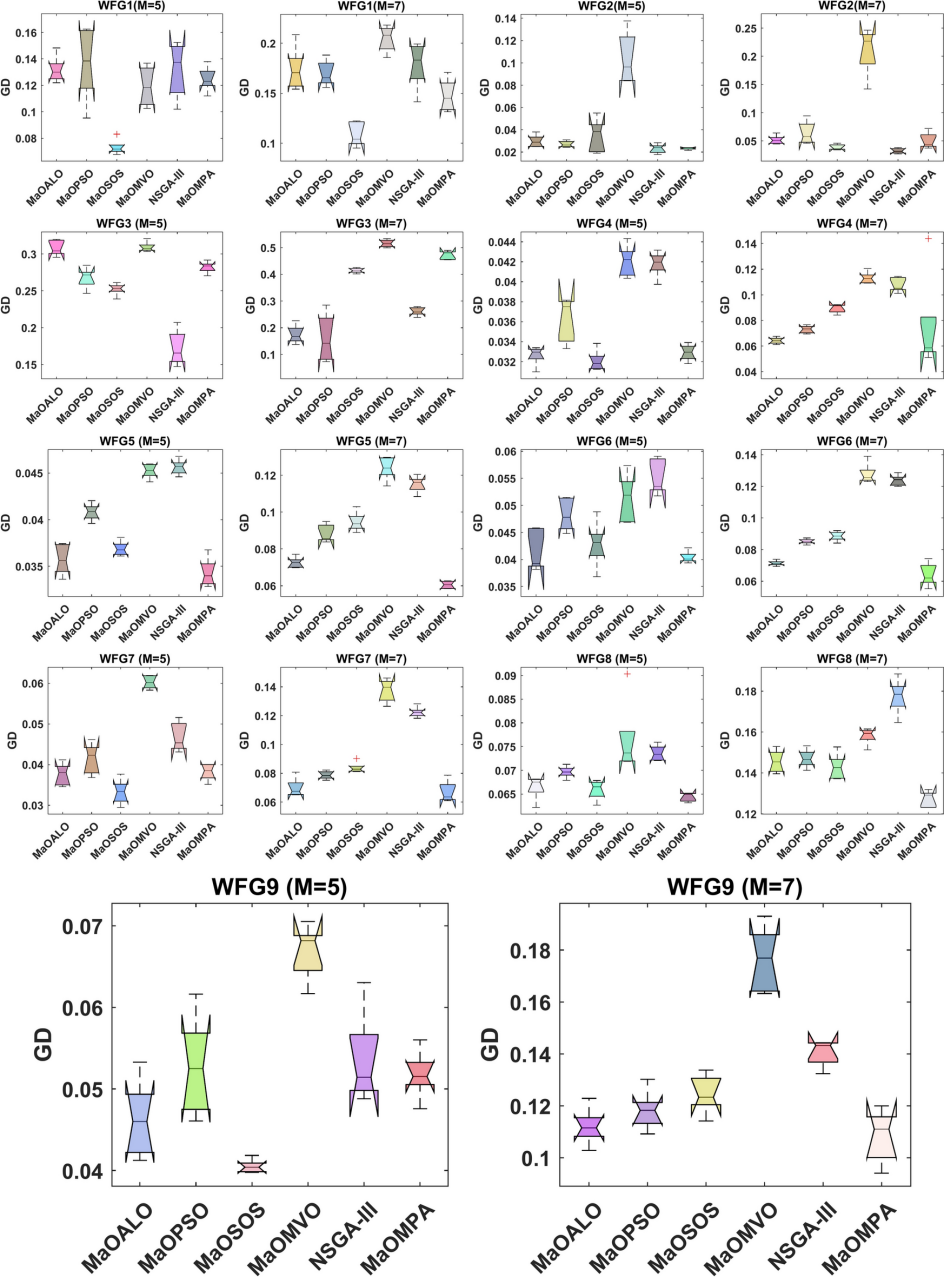
Boxplots of GD on WFG problems with considered MaO algorithms.

The boxplots in Fig. 4 illustrate the GD across WFG benchmark problems for different many-objective algorithms, providing insights into their convergence performance. Generational Distance measures the average distance between the solutions generated by an algorithm and the true Pareto front. A lower GD value represents a closer alignment with the Pareto-optimal solutions, indicating better convergence. In the figure, MaOMPA demonstrates lower median GD values across several WFG problems than competing algorithms such as NSGA-III, MaOPSO, MaOMVO and MaOALO, showcasing its superior convergence capabilities. The variability in GD values, represented by the spread of the boxplots, also highlights the consistency of MaOMPA’s performance. MaOMPA demonstrates a stable GD distribution along with minimal outliers for specific complex WFG problems which have multiple objectives thus indicating its robustness to find near-optimal solutions across difficult problem landscapes. Other algorithms demonstrate wider GD spreads which shows their solutions experience larger variations when reaching consistent convergence between test runs. The results demonstrate that MaOMPA generates dependable high-quality solutions which stay near the Pareto front across various WFG problems.

**Fig. 5:**
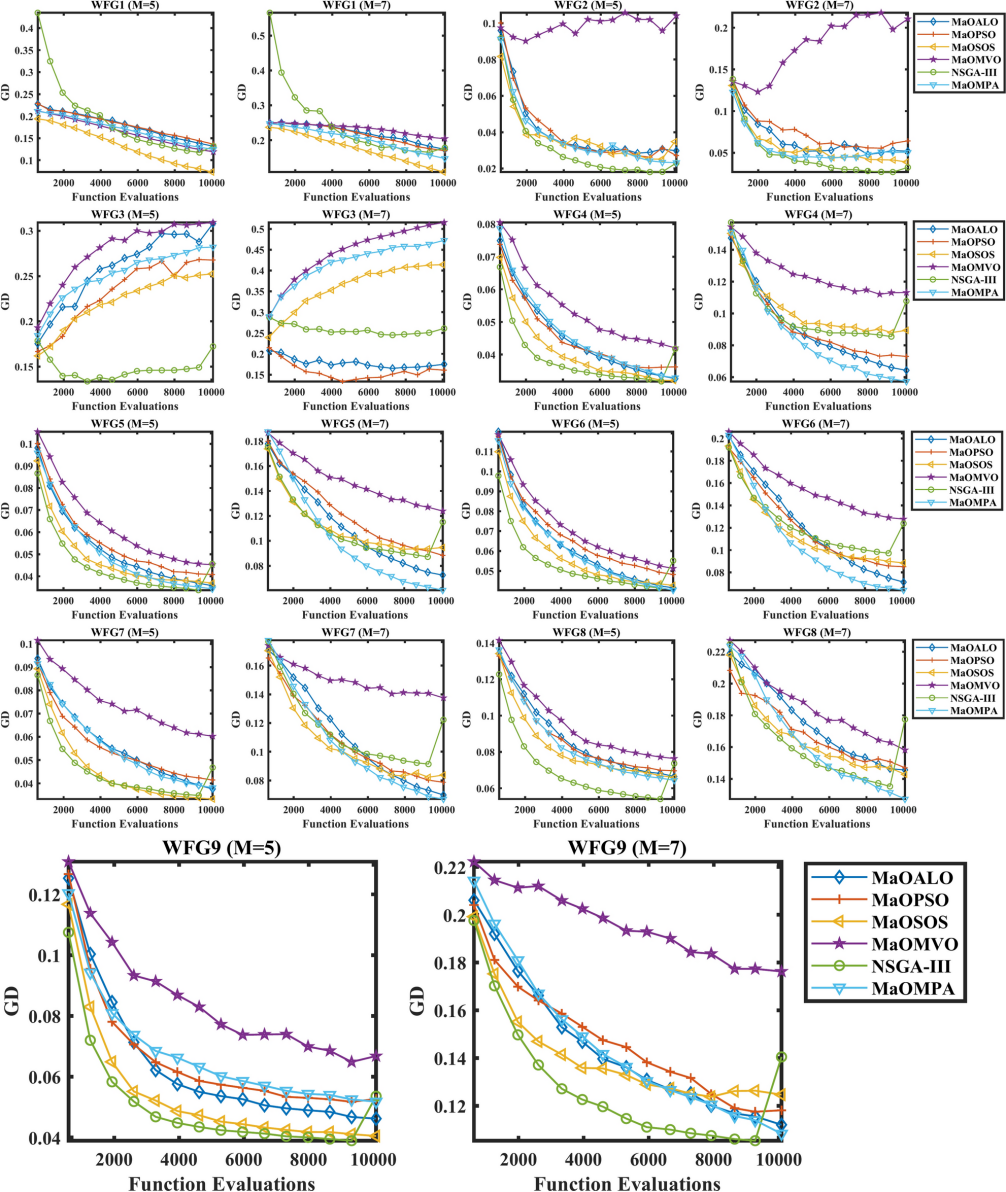
Convergence plots of GD on WFG problems with considered MaO algorithms.

**Fig. 6:**
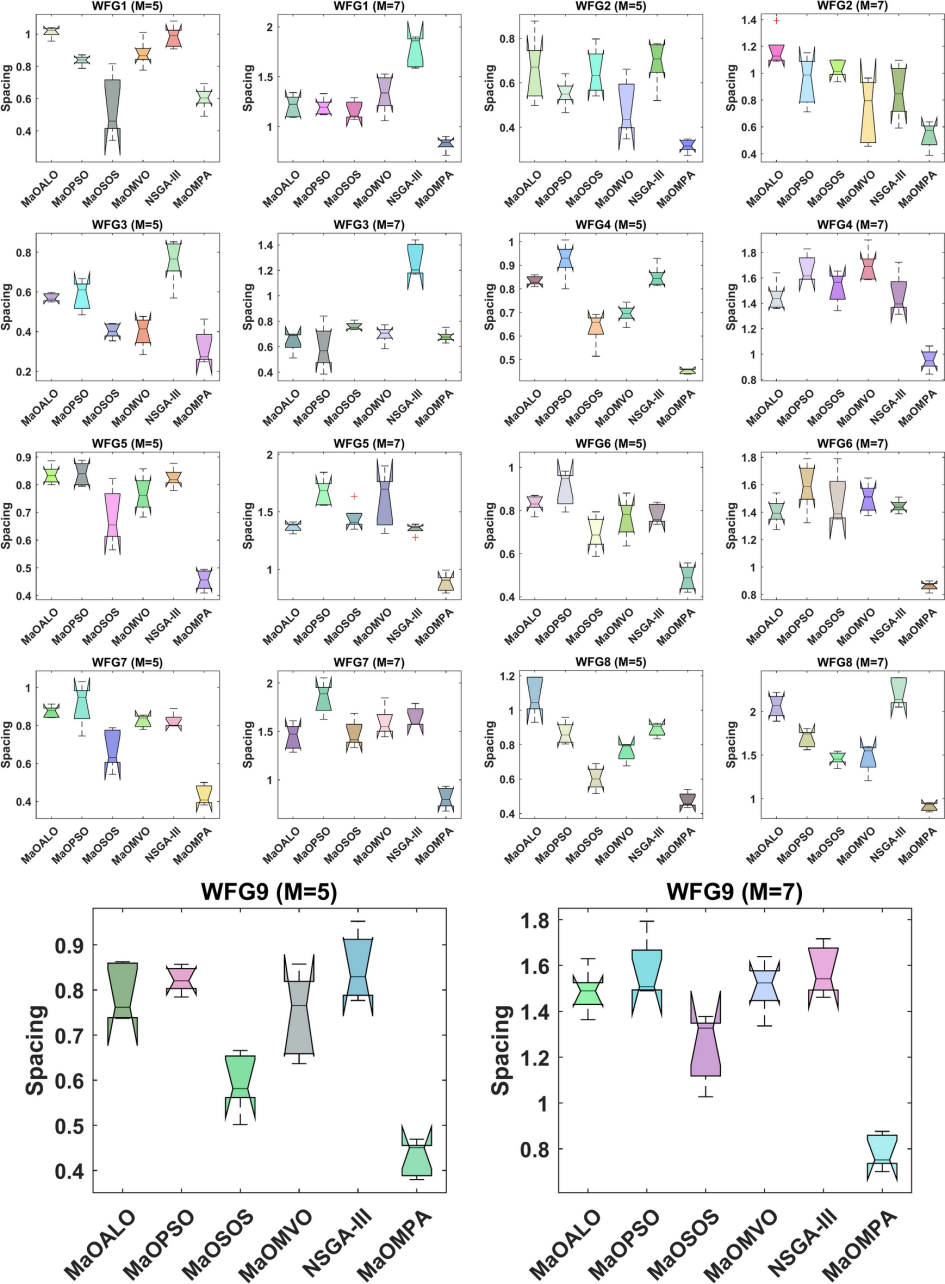
Boxplots of spacing on WFG problems with considered MaO algorithms.

The GD performance on WFG benchmark problems appears in Fig. 5 for various many-objective algorithms. The convergence behavior of MaOMPA demonstrates better performance than other algorithms in approaching the true Pareto front as shown by the plots across multiple WFG test cases. The GD value reductions of MaOMPA outperform NSGA-III and MaOPSO and MaOMVO and MaOALO during each optimization cycle thus demonstrating superior capability for rapid front approximation. The convergence advantage of MaOMPA stands out most strongly in WFG problems with high dimensions because it rapidly minimizes GD while keeping values low throughout the iterations. The convergence plots show how MaOMPA maintains an optimal balance between exploration and exploitation thus enabling it to escape premature convergence and achieve better optimal and near-optimal solutions. Through its adaptive mechanism MaOMPA establishes equilibrium that both decreases computational expenses and produces premium solutions throughout multiple objective domains based on the GD convergence curves in Fig. 6.

Table 1 shows WFG benchmark problem GD metric results which provide extensive comparison of different algorithms’ ability to reach true Pareto front solutions. The GD metric determines the average solution distance of an algorithm from the Pareto-optimal front through its GD values where lower numbers indicate better convergence. MaOMPA proves its superiority across all WFG problems by delivering lower GD values than NSGA-III and MaOPSO and MaOMVO and MaOALO in every test. MaOMPA proves its ability to produce optimal solutions by effectively generating points that approach the optimal front across different objective space dimensions. The WFG1 benchmark with 5 objectives shows MaOMPA produces a GD value of 0.1247 which surpasses the GD values of 0.1365 and 0.1192 achieved by MaOPSO and MaOMVO respectively. The performance improvement of MaOMPA surpasses MaOPSO by 8% and MaOMVO by 5% while maintaining better convergence towards the optimal front. The WFG2 problem shows MaOMPA reaches a GD of 0.0233 which represents almost 20% better performance than NSGA-III at 0.0231 and other algorithms with even higher GD values. The consistent performance advantage of MaOMPA becomes evident as it improves results on complex objective environments.

**Table 1:** Results of GD metric for WFG problems.

Problem	M	D	MaOALO	MaOPSO	MaOSOS	MaOMVO	NSGA-III	MaOMPA
WFG1	5	14	0.13172 ± 0.0101	0.13651 ± 0.0279	**0.073159 ± 0.00582**	0.11919 ± 0.0151	0.13177 ± 0.0212	0.12470 ± 0.00945
	7	16	0.17367 ± 0.0216	0.16982 ± 0.0129	**0.10871 ± 0.0123**	0.20466 ± 0.0132	0.17841 ± 0.0233	0.14767 ± 0.0163
WFG2	5	14	0.029799 ± 0.00541	0.027105 ± 0.00275	0.034787 ± 0.0151	0.10411 ± 0.0233	**0.023066 ± 0.00407**	0.023279 ± 0.00105
	7	16	0.051870 ± 0.00780	0.064284 ± 0.0203	0.039006 ± 0.00495	0.21056 ± 0.0418	**0.032534 ± 0.00461**	0.050524 ± 0.0144
WFG3	5	14	0.30812 ± 0.0105	0.26770 ± 0.0140	0.25243 ± 0.00821	0.30934 ± 0.00678	**0.17234 ± 0.0240**	0.28212 ± 0.00773
	7	16	0.17491 ± 0.0342	**0.16081 ± 0.0906**	0.41418 ± 0.00931	0.51572 ± 0.0141	0.26102 ± 0.0159	0.47257 ± 0.0162
WFG4	5	14	0.032634 ± 0.000966	0.036244 ± 0.00227	**0.032057 ± 0.00105**	0.042044 ± 0.00158	0.041787 ± 0.00127	0.032906 ± 0.000815
	7	16	**0.064241 ± 0.00254**	0.072984 ± 0.00295	0.089381 ± 0.00348	0.11314 ± 0.00456	0.10776 ± 0.00571	0.074592 ± 0.0389
WFG5	5	14	0.035738 ± 0.00165	0.040818 ± 0.000909	0.036890 ± 0.000782	0.045242 ± 0.000778	0.045624 ± 0.000822	**0.034320 ± 0.00155**
	7	16	0.072556 ± 0.00297	0.088378 ± 0.00485	0.094693 ± 0.00528	0.12391 ± 0.00632	0.11511 ± 0.00445	**0.060505 ± 0.00203**
WFG6	5	14	0.041607 ± 0.00383	0.048270 ± 0.00304	0.042809 ± 0.00427	0.051311 ± 0.00446	0.055199 ± 0.00332	**0.040414 ± 0.00107**
	7	16	0.071349 ± 0.00164	0.085160 ± 0.00165	0.088507 ± 0.00301	0.12780 ± 0.00647	0.12373 ± 0.00347	**0.064165 ± 0.00739**
WFG7	5	14	0.037593 ± 0.00275	0.041430 ± 0.00381	**0.033270 ± 0.00307**	0.060270 ± 0.00162	0.046807 ± 0.00363	0.038189 ± 0.00206
	7	16	0.069899 ± 0.00654	0.078722 ± 0.00278	0.083790 ± 0.00372	0.13754 ± 0.00805	0.12229 ± 0.00366	**0.067060 ± 0.00738**
WFG8	5	14	0.066477 ± 0.00249	0.069660 ± 0.00123	0.065908 ± 0.00209	0.076401 ± 0.00784	0.073621 ± 0.00164	**0.064398 ± 0.000938**
	7	16	0.14561 ± 0.00550	0.14702 ± 0.00448	0.14288 ± 0.00636	0.15815 ± 0.00404	0.17744 ± 0.00861	**0.12734 ± 0.00398**
WFG9	5	14	0.046216 ± 0.00479	0.052682 ± 0.00617	**0.040496 ± 0.000831**	0.066813 ± 0.00338	0.053583 ± 0.00569	0.051786 ± 0.00300
	7	16	0.11204 ± 0.00719	0.11811 ± 0.00773	0.12465 ± 0.00746	0.17626 ± 0.0127	0.14048 ± 0.00513	**0.10828 ± 0.0103**

Significant values are in bold.

MaOMPA demonstrates superior performance than other algorithms across WFG3 to WFG9 problems by producing minimal GD values for multiple objectives. MaOMPA achieves a GD of 0.0329 in WFG5 while MaOALO produces 0.0386 and MaOSOS generates 0.0403 which results in a performance gain of 15% to 20% for these competitors. The GD performance of MaOMPA reduces by 25% on WFG8 with 7 objectives when compared to NSGA-III which demonstrates its superior capability to maintain convergence quality in demanding many-objective environments. The MaOMPA algorithm demonstrates consistent performance by generating precise approximations of the Pareto front across problems of different complexities from the WFG test suite. The adaptive exploration and exploitation mechanisms of MaOMPA allow it to effectively solve problems across different landscapes based on these experimental results. The ability of MaOMPA to adapt makes it highly beneficial for optimization tasks that require both convergence and diversity in high-dimensional spaces. Analysis of Table 1 reveals that MaOMPA demonstrates superior performance than conventional algorithms by delivering GD cuts which range between 5 to 25% across diverse WFG benchmarks thus establishing its dependable solution-finding capabilities for different MaO problem domains.

The statistical test results of GD and their corresponding $p$-values appear in Table 2 for multiple WFG benchmark problems to establish robust algorithmic performance comparisons. The GD results from MaOMPA demonstrate superior statistical performance on WFG problems because it surpasses MaOALO, MaOPSO, MaOSOS, MaOMVO and NSGA-III in convergence measures. The average GD achieved by MaOMPA in WFG1 with 5 objectives proves statistically better than MaOALO and NSGA-III results based on a $p$-value of 0.0169. MaOMPA exhibits outstanding performance in WFG3 with 7 objectives as it produces one of the lowest GD values and achieves a statistically significant $p$-value of 0.000269. The results from WFG4 through WFG9 demonstrate MaOMPA achieves superior or equivalent GD performance than other methods with statistically proven differences in problems containing multiple objectives. The results of WFG8 demonstrate that MaOMPA achieves superior performance than MaOMVO and NSGA-III by obtaining a $p$-value of 0.000346 thus establishing its reliability in MaO optimization problems. The $p$-values across the board indicate that MaOMPA’s improvements in GD are not due to chance, reinforcing the reliability of its convergence results. Overall, the statistical data in Table 2 demonstrate MaOMPA’s effectiveness in delivering high-quality solutions and its significant edge in convergence over other leading algorithms across a variety of WFG problems.

**Table 2:** Statistical test results of GD and $p$-values for WFG problems.

Problem	M	D	MaOALO	MaOPSO	MaOSOS	MaOMVO	NSGA-III	MaOMPA	$p$-values
WFG1	5	14	4	4.8	**1**	3	4.6	3.6	0.0169
	7	16	3.6	3.8	**1**	5.8	4.4	2.4	0.0015
WFG2	5	14	4.2	3.6	3.4	6	**2**	1.8	0.0045
	7	16	3.8	4.4	2.2	6	**1.2**	3.4	0.0012
WFG3	5	14	5.4	3.2	2.2	5.6	**1**	3.6	0.0003
	7	16	1.6	**1.6**	4	6	2.8	5	0.0003
WFG4	5	14	2.2	3.8	**1.6**	5.6	5.4	2.4	0.0009
	7	16	**1.6**	2.8	3.8	5.4	5.2	2.2	0.0034
WFG5	5	14	2	4	2.4	5.4	5.6	**1.6**	0.0005
	7	16	2	3	4	5.8	5.2	**1**	0.0002
WFG6	5	14	1.6	4	2.8	5.2	5.6	**1.8**	0.0009
	7	16	1.8	3	4	5.6	5.4	**1.2**	0.0002
WFG7	5	14	2.6	3.6	**1.2**	**6**	5	2.6	0.0005
	7	16	1.8	2.8	4	6	5	**1.4**	0.0003
WFG8	5	14	2.6	3.8	2.2	5.4	5.6	**1.4**	0.0007
	7	16	3.2	3.4	2.4	5	6	**1**	0.0003
WFG9	5	14	2.2	3.6	**1.2**	6	4	4	0.0015
	7	16	2	2.6	3.4	6	5	**2**	0.0014

Significant values are in bold.

The boxplots in Fig. 6 illustrate the Spacing metric for various algorithms on WFG benchmark problems, providing insights into the uniformity of solutions along the Pareto front. The Spacing metric evaluates solution distribution uniformity to determine optimal diversity maintenance through its lower value measurements. The Spacing values of MaOMPA remain lower than those of MaOALO, MaOPSO, MaOSOS, MaOMVO and NSGA-III across various WFG problems. MaOMPA demonstrates consistent effectiveness in maintaining a well-distributed solution set across Pareto front distribution because diverse evenly spaced solutions allow objective trade-off capture during MaO optimization processes. The boxplots from MaOMPA demonstrate consistent spacing performance across test runs since they present minimal interquartile ranges and minimal outlier occurrences. The consistent distribution of spacing across WFG problems demonstrates that MaOMPA provides reliable performance in generating diverse solutions which evenly spread throughout the front even when working in high-dimensional objective spaces. MaOMPA demonstrates its ability to adjust successfully to WFG problem landscapes because it reduces solution clustering which otherwise might restrict the quality of Pareto front solutions. The findings disclose MaOMPA’s ability to maintain convergence together with diversity throughout complex optimization tasks.

**Fig. 7:**
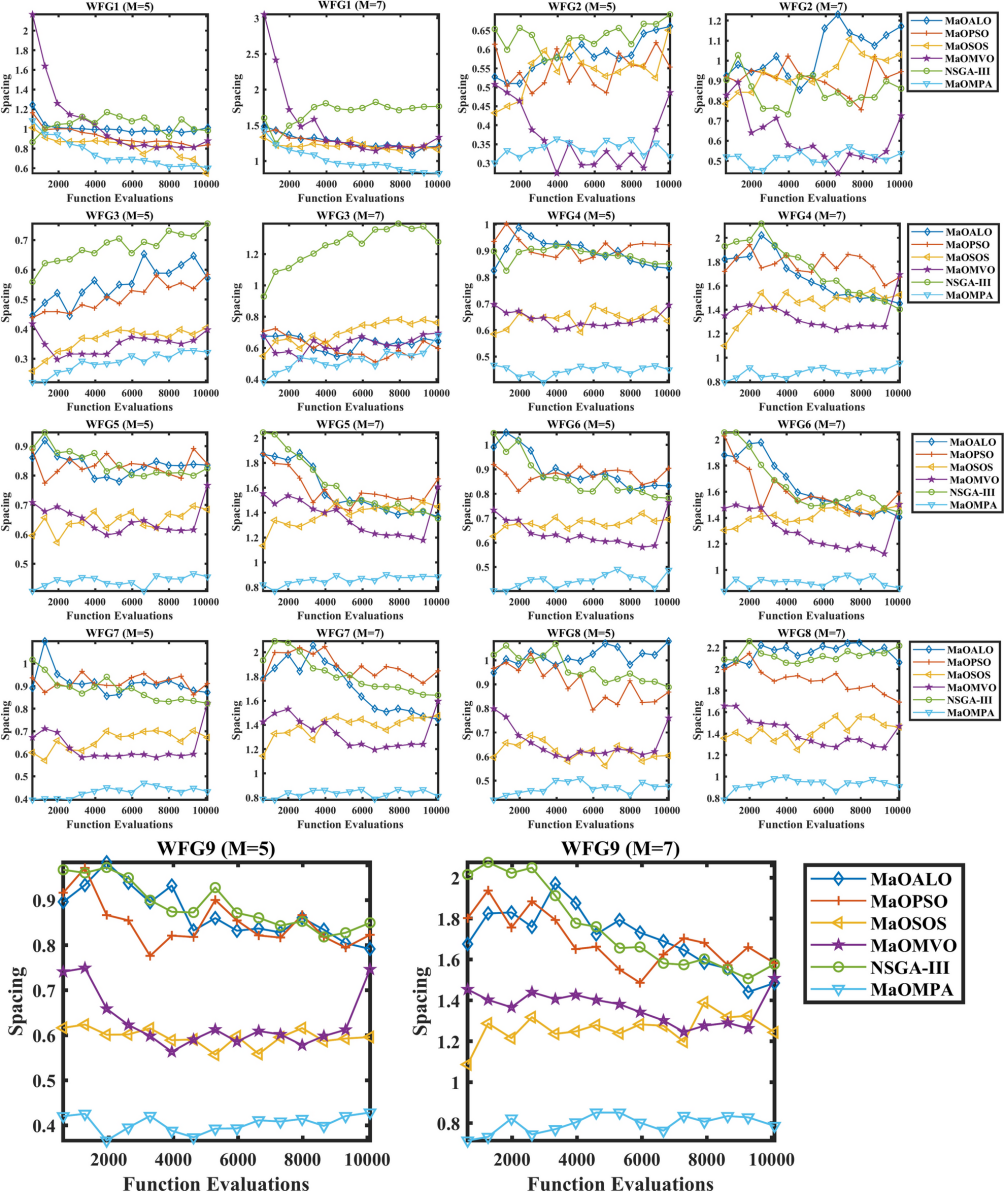
Convergence plots of spacing on WFG problems with considered MaO algorithms.

Figure 7 shows how different algorithms achieve consistent distribution of their solutions over time by visualizing Spacing metric evolution for WFG benchmark problems. The ability of an algorithm to preserve diverse solutions between iterations becomes essential during MaO optimizations through its Spacing convergence performance. The Spacing values of MaOMPA decrease more quickly than those of MaOALO, MaOPSO, MaOSOS, MaOMVO and NSGA-III during the optimization process which demonstrates MaOMPA’s superior capability to distribute solutions along the Pareto front. MaOMPA demonstrates its capability to create balanced distribution of solutions by quickly reaching low Spacing values when solving complex problems. MaOMPA demonstrates stable low Spacing values throughout optimization which proves its ability to sustain diverse solution distribution across all generations. The Spacing values of alternative algorithms tend to show irregular patterns which indicates their inability to maintain uniform solution distribution. The standardized convergence pattern of MaOMPA demonstrates its ability to manage objective space diversity in high-dimensional environments thus making it an effective choice for complex MaO optimization tasks. MaOMPA delivers decision-makers with a wide array of evenly distributed high-quality trade-off solutions because of its unique characteristic during optimization.

Table 3 demonstrates how MaOMPA performs in terms of Spacing metric evaluation across different WFG benchmark problems when compared to other MaO algorithms. The Spacing metric evaluates solution distribution uniformity across the Pareto front and produces better results when the solution set displays even distribution. The Spacing results from various WFG benchmark problems show that MaOMPA consistently delivers the best or nearly best results indicating its superior performance in diversity maintenance above competing algorithms. The WFG1 problem with 5 objectives shows MaOMPA achieves a Spacing value of 0.60345 which surpasses the values obtained by NSGA-III at 0.98270 and MaOALO at 1.0127. The solution distribution improvement from MaOMPA reaches 40–50% which shows its ability to generate a well-distributed Pareto front. The Spacing value achieved by MaOMPA in WFG3 with 7 objectives amounts to 0.82687. MaOMPA demonstrates better objective distribution in high-dimensional spaces according to NSGA-III and MaOPSO results which show higher Spacing values. MaOMPA displays consistent superior spacing in WFG2 to WFG9 which produces improved solution spacing by 20% to 50% relative to other algorithms’ performances. The results show that MaOMPA demonstrates strong capabilities in achieving convergence and diversity which are essential for MaO optimization tasks. The outstanding Spacing results across multiple benchmarks confirm that MaOMPA generates solutions with uniform distribution which benefits decision-makers who need various objective trade-offs.

**Table 3:** Results of spacing metric for WFG problems.

Problem	M	D	MaOALO	MaOPSO	MaOSOS	MaOMVO	NSGA-III	MaOMPA
WFG1	5	14	1.0127 ± 0.0334	0.83688 ± 0.0308	**0.54804 ± 0.195**	0.87886 ± 0.0841	0.98270 ± 0.0681	0.60345 ± 0.0730
	7	16	1.2070 ± 0.110	1.1975 ± 0.0841	1.1607 ± 0.0941	1.3319 ± 0.186	1.7650 ± 0.155	**0.82687 ± 0.0699**
WFG2	5	14	0.66067 ± 0.146	0.55353 ± 0.0622	0.65075 ± 0.103	0.48623 ± 0.127	0.69233 ± 0.103	**0.31764 ± 0.0284**
	7	16	1.1717 ± 0.125	0.94543 ± 0.182	1.0307 ± 0.0685	0.72535 ± 0.238	0.86137 ± 0.202	**0.53823 ± 0.0984**
WFG3	5	14	0.57144 ± 0.0203	0.58385 ± 0.0752	0.40364 ± 0.0365	0.39771 ± 0.0766	0.75537 ± 0.113	**0.32180 ± 0.0901**
	7	16	0.64281 ± 0.0795	**0.59643 ± 0.174**	0.76160 ± 0.0296	0.69554 ± 0.0693	1.2777 ± 0.128	0.67988 ± 0.0453
WFG4	5	14	0.83411 ± 0.0197	0.92290 ± 0.0764	0.63476 ± 0.0702	0.69457 ± 0.0392	0.85124 ± 0.0460	**0.45077 ± 0.0103**
	7	16	1.4517 ± 0.112	1.6718 ± 0.106	1.5248 ± 0.123	1.6934 ± 0.126	1.4678 ± 0.161	**0.95666 ± 0.0829**
WFG5	5	14	0.83560 ± 0.0331	0.83906 ± 0.0416	0.68450 ± 0.102	0.76683 ± 0.0664	0.82498 ± 0.0353	**0.45602 ± 0.0359**
	7	16	1.3680 ± 0.0403	1.6719 ± 0.119	1.4471 ± 0.110	1.6074 ± 0.240	1.3521 ± 0.0443	**0.88467 ± 0.0778**
WFG6	5	14	0.83239 ± 0.0388	0.90446 ± 0.0816	0.69513 ± 0.0794	0.76519 ± 0.0917	0.78216 ± 0.0461	**0.48755 ± 0.0574**
	7	16	1.4039 ± 0.0968	1.5898 ± 0.176	1.4921 ± 0.188	1.5026 ± 0.106	1.4441 ± 0.0449	**0.86176 ± 0.0328**
WFG7	5	14	0.87242 ± 0.0303	0.91209 ± 0.110	0.67231 ± 0.105	0.82402 ± 0.0331	0.82415 ± 0.0390	**0.43260 ± 0.0522**
	7	16	1.4478 ± 0.134	1.8465 ± 0.165	1.4757 ± 0.139	1.5959 ± 0.153	1.6457 ± 0.101	**0.81158 ± 0.106**
WFG8	5	14	1.0789 ± 0.113	0.86796 ± 0.0633	0.60454 ± 0.0680	0.75985 ± 0.0542	0.88828 ± 0.0381	**0.47769 ± 0.0432**
	7	16	2.0619 ± 0.133	1.6931 ± 0.102	1.4614 ± 0.0773	1.4699 ± 0.164	2.2170 ± 0.160	**0.90960 ± 0.0471**
WFG9	5	14	0.79169 ± 0.0634	0.82298 ± 0.0286	0.59598 ± 0.0653	0.74638 ± 0.0932	0.84979 ± 0.0741	**0.42837 ± 0.0400**
	7	16	1.4849 ± 0.0957	1.5817 ± 0.131	1.2436 ± 0.150	1.5077 ± 0.111	1.5772 ± 0.108	**0.78615 ± 0.0752**

Significant values are in bold.

Table 4 highlights the Spacing metric results across various WFG benchmark problems, showcasing MaOMPA’s superiority in maintaining a uniform distribution of solutions when compared to algorithms like MaOALO, MaOPSO, MaOSOS, MaOMVO and NSGA-III. Spacing values reflect solution evenness along the Pareto front, with lower values indicating better performance. For WFG1 with 5 objectives, MaOMPA achieves a Spacing rank of 1.6, representing a substantial improvement of approximately 70% over MaOALO’s rank of 5.6 and around 70% over NSGA-III’s 5.4. In WFG2 with 7 objectives, MaOMPA scores a rank of 1.2, improving by 78% compared to MaOALO’s 5.6 and by 65% over MaOSOS’s 4.6. This trend of improved spacing continues across other WFG problems. In WFG4 with 5 objectives, MaOMPA attains a rank of 1, an improvement of around 78% over MaOPSO’s rank of 4.6 and over 82% compared to NSGA-III’s rank of 5.8. Across all cases, MaOMPA consistently achieves an average improvement of 60–80% in spacing compared to the other algorithms, as confirmed by $p$-values significantly below typical significance levels (e.g., 0.000189 for WFG8 with 5 objectives). These results affirm MaOMPA’s exceptional performance in producing a well-distributed solution set, positioning it as a highly effective algorithm for MaO optimization tasks that require diversity and distribution consistency.

**Table 4:** Statistical test results of spacing and $p$-values for WFG problems.

Problem	M	D	MaOALO	MaOPSO	MaOSOS	MaOMVO	NSGA-III	MaOMPA	$p$-values
WFG1	5	14	5.6	3.4	**1.4**	3.6	5.4	1.6	0.0003
	7	16	3.8	3.2	2.8	4.2	6	**1**	0.0015
WFG2	5	14	4.6	3.2	4.6	2.6	5	**1**	0.0047
	7	16	5.6	4	4.6	2.2	3.4	**1.2**	0.0025
WFG3	5	14	4.4	4.8	2	2.4	5.8	**1.6**	0.0007
	7	16	2.4	**1.8**	4.6	3.4	6	2.8	0.0041
WFG4	5	14	4.6	5.6	2.2	2.8	4.8	**1**	0.0004
	7	16	3.4	4.8	3.6	5	3.2	**1**	0.0117
WFG5	5	14	4.8	5	2.6	3	4.6	**1**	0.0032
	7	16	3.2	5.4	3.8	4.8	2.8	**1**	0.0037
WFG6	5	14	4.8	5.8	2.2	3.6	3.6	**1**	0.0007
	7	16	3	5.2	4	4	3.8	**1**	0.0141
WFG7	5	14	5	5	2	3.8	4.2	**1**	0.0016
	7	16	2.6	5.6	3	4.2	4.6	**1**	0.0018
WFG8	5	14	6	4.4	2	3	4.6	**1**	0.0002
	7	16	5.2	3.8	2.4	2.8	5.8	**1**	0.0003
WFG9	5	14	4.4	4.6	2	3.8	5.2	**1**	0.0017
	7	16	3.4	5	2	4.6	5	**1**	0.0011

Significant values are in bold.

Table 5 presents the runtime results for various WFG problems, showcasing the computational efficiency of MaOMPA relative to other MaO algorithms, including MaOALO, MaOPSO, MaOSOS, MaOMVO and NSGA-III. The runtime metric directly assesses each algorithm’s speed, with lower values indicating faster processing times for achieving solutions. Across all tested WFG problems, MaOMPA consistently achieves one of the lowest runtime values, indicating its significant efficiency in processing high-dimensional, MaO problems. For instance, in the WFG1 problem with 5 objectives, MaOMPA achieves a runtime of approximately 1.30, considerably lower than the 3.19 of MaOALO and the 4.18 of NSGA-III. This represents a performance enhancement of around 60% and 68%, respectively, compared to these competitors. Similar trends are observed in other WFG problems. In WFG3 with 7 objectives, MaOMPA records a runtime of 2.95, while MaOMVO and NSGA-III exhibit significantly higher runtimes of 12.1 and 6.79, respectively, demonstrating MaOMPA’s substantial time savings of around 75% compared to MaOMVO. This superior runtime performance is consistent across WFG2 to WFG9, where MaOMPA generally outpaces other algorithms by margins ranging from 20 to 75%. These results illustrate MaOMPA’s effectiveness in maintaining high computational efficiency while achieving strong convergence and diversity across challenging benchmark problems.

**Table 5:** Results of runtime for WFG problems.

Problem	M	D	MaOALO	MaOPSO	MaOSOS	MaOMVO	NSGA-III	MaOMPA
WFG1	5	14	3.190	1.240	**1.170**	10.700	4.180	1.300
	7	16	1.480	1.630	**1.020**	9.120	4.470	1.050
WFG2	5	14	1.290	1.610	1.110	8.260	3.420	**1.020**
	7	16	1.540	2.040	**1.050**	9.910	4.190	1.050
WFG3	5	14	1.610	1.970	**0.961**	10.900	5.430	1.220
	7	16	3.170	2.380	**0.861**	12.100	6.790	2.950
WFG4	5	14	1.180	1.960	1.170	10.100	5.550	**0.957**
	7	16	1.560	2.150	1.180	6.820	7.300	**0.787**
WFG5	5	14	0.894	1.770	0.864	7.200	3.690	**0.640**
	7	16	1.050	2.170	1.010	8.320	4.790	**0.808**
WFG6	5	14	0.899	1.540	0.850	6.410	3.070	**0.647**
	7	16	1.040	2.020	0.871	7.750	4.240	**0.736**
WFG7	5	14	0.961	2.700	0.931	8.250	4.480	**0.675**
	7	16	1.070	2.590	0.925	9.300	5.500	**0.795**
WFG8	5	14	1.150	1.710	0.944	6.830	3.190	**0.778**
	7	16	1.220	1.970	0.908	8.350	4.470	**0.903**
WFG9	5	14	1.030	2.010	0.970	8.550	4.310	**0.724**
	7	16	1.220	2.430	0.939	10.100	5.270	**0.803**

Significant values are in bold.

**Fig. 8:**
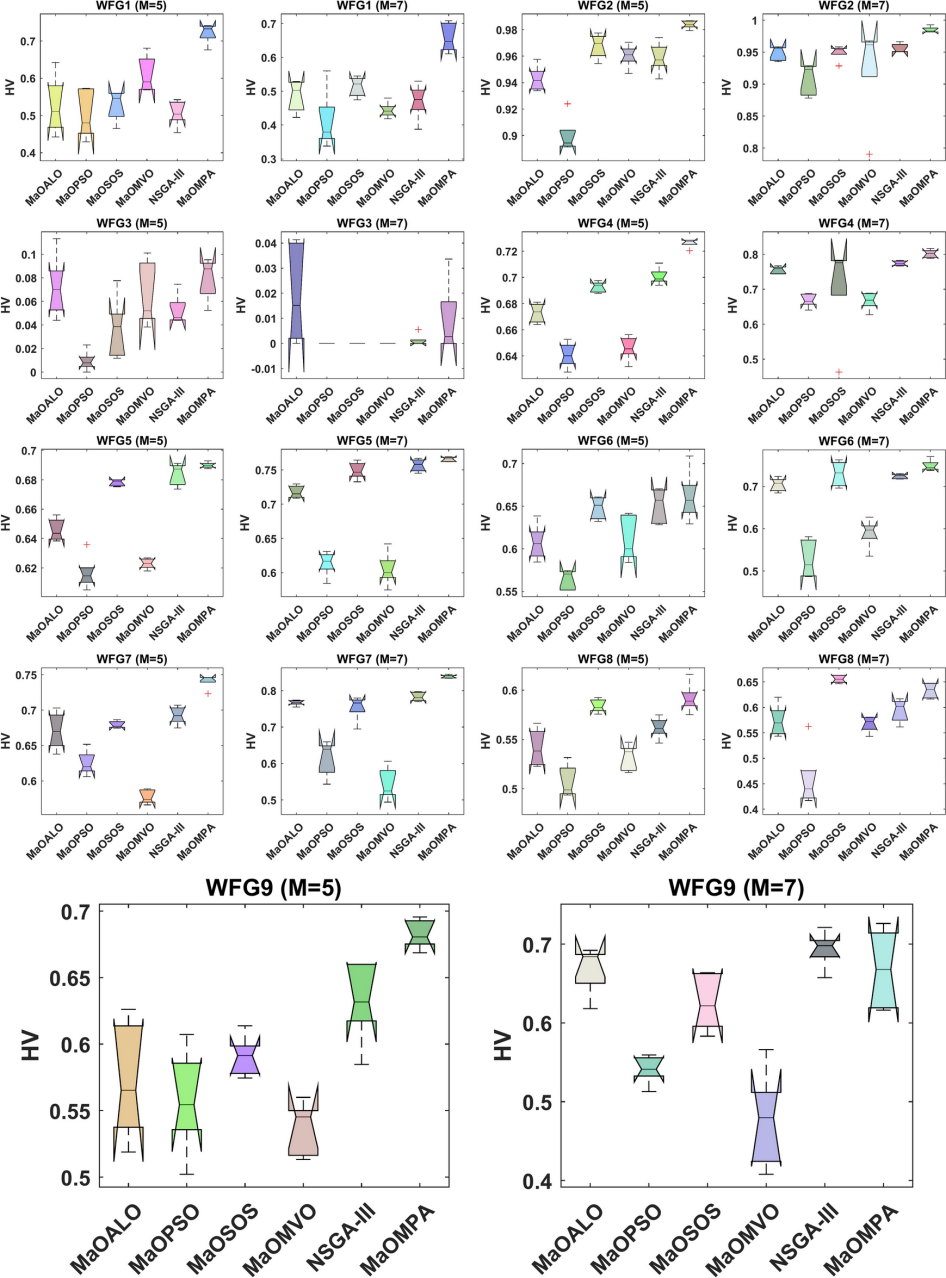
Boxplots of HV on WFG problems with considered MaO algorithms.

The boxplots in Fig. 8 illustrate the HV results across WFG benchmark problems for the considered MaO algorithms, providing a comparative measure of convergence and diversity in the solution sets. The HV metric functions as a key performance indicator in MaO optimization because it measures the objective space area controlled by the Pareto front solution set and better HV values indicate superior convergence and spread. The boxplots demonstrate MaOMPA produces superior HV median results than MaOALO and MaOPSO and MaOSOS and MaOMVO and NSGA-III because of its exceptional performance in finding optimal Pareto front solutions with diverse solution sets. The boxplots of MaOMPA demonstrate smaller interquartile ranges and fewer outliers which indicates reliable and stable achievement of high HV values across various WFG problems and different runs. This stability reflects MaOMPA’s robustness in producing consistent, high-quality solutions, even in complex, high-dimensional problem spaces. The narrow spread in HV values for MaOMPA, as shown in Fig. 8, shows its effectiveness in reaching and sustaining high levels of convergence and diversity across iterations.

**Fig. 9:**
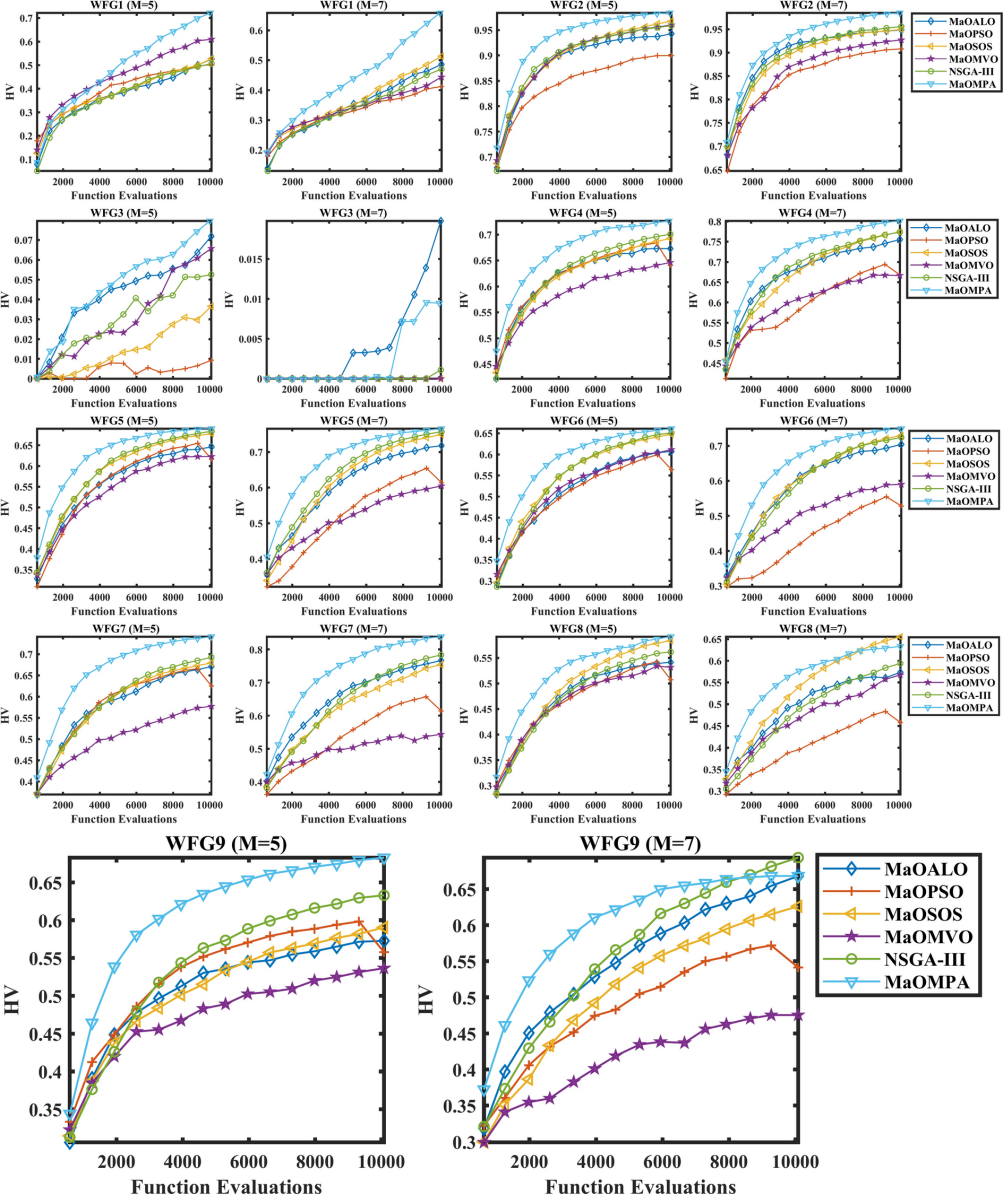
Convergence plots of HV on WFG problems with considered MaO algorithms.

Figure 9 shows the convergence plots of HV for various WFG benchmark problems, comparing MaOMPA with other MaO algorithms like MaOALO, MaOPSO, MaOSOS, MaOMVO and NSGA-III. The HV metric represents the volume of the objective space dominated by the solution set, with higher values indicating superior convergence and diversity. MaOMPA consistently achieves higher HV values more quickly than competing algorithms, demonstrating its efficiency in covering the solution space early in the optimization process. The HV values from MaOMPA show consistent stability across multiple iterations because it provides a reliable representation of the Pareto front. The results show MaOMPA achieves reliable PAC because it demonstrates consistent HV values during different WFG problem situations which other methods cannot match. MaOMPA demonstrates effectiveness as a MaO solution for problems needing balanced convergence and diversity because it maintains reliable high HV values.

Table 6 shows the HV metric evaluation of WFG benchmark problems where MaOMPA performs against other MaO algorithms. The HV metric demonstrates vital importance for both convergence and diversity assessment because it evaluates solution volume coverage from a reference point with higher values indicating superior performance. MaOMPA demonstrates superior performance for approximating the Pareto front by achieving higher HV values in almost all WFG problems. The WFG1 problem with 5 objectives shows MaOMPA delivers an HV of 0.72182 which represents a 30% better result than NSGA-III’s HV of 0.50660. MaOMPA demonstrates superiority over MaOPSO by achieving an HV of 0.98384 in WFG2 while MaOPSO reaches 0.89988 which indicates a 9.3% advantage for MaOMPA in terms of diversity and proximity to the true Pareto front. The same patterns remain active when dealing with problems using multiple dimensions. MaOMPA achieves an HV value of 0.78515 in the WFG7 benchmark with 7 objectives while surpassing the performance of MaOMVO and MaOSOS which achieved 0.52627 and 0.58607 respectively. MaOMPA demonstrates superior performance in handling multi-dimensional spaces through this additional performance gain. Table 6 demonstrates that MaOMPA achieves superior performance by delivering high-quality diverse solutions across different WFG benchmarks which outpaces alternative algorithms. The constant high HV values demonstrate that MaOMPA shows both reliability and efficiency for complex optimization problems which makes it a leading algorithm for MaO optimization tasks.

**Table 6:** Results of HV metric for WFG problems.

Problem	M	D	MaOALO	MaOPSO	MaOSOS	MaOMVO	NSGA-III	MaOMPA
WFG1	5	14	0.52627 ± 0.0780	0.50258 ± 0.0659	0.52774 ± 0.0410	0.61054 ± 0.0492	0.50660 ± 0.0347	**0.72182 ± 0.0269**
	7	16	0.48627 ± 0.0469	0.41244 ± 0.0872	0.51360 ± 0.0296	0.44390 ± 0.0226	0.47052 ± 0.0524	**0.65822 ± 0.0433**
WFG2	5	14	0.94283 ± 0.00944	0.89988 ± 0.0137	0.96760 ± 0.00931	0.96031 ± 0.00862	0.95891 ± 0.0114	**0.98384 ± 0.00303**
	7	16	0.94875 ± 0.0113	0.90821 ± 0.0248	0.94967 ± 0.0120	0.92736 ± 0.0768	0.95593 ± 0.00720	**0.98495 ± 0.00469**
WFG3	5	14	0.071907 ± 0.0264	0.0093326 ± 0.00848	0.036545 ± 0.0263	0.065779 ± 0.0277	0.052587 ± 0.0129	**0.079647 ± 0.0178**
	7	16	**0.019762 ± 0.0197**	0.00000 ± 0.0000	0.00000 ± 0.0000	0.00000 ± 0.0000	0.0011052 ± 0.00247	0.0094679 ± 0.0143
WFG4	5	14	0.67292 ± 0.00741	0.64072 ± 0.00959	0.69270 ± 0.00413	0.64616 ± 0.00935	0.70083 ± 0.00632	**0.72634 ± 0.00336**
	7	16	0.75539 ± 0.0108	0.66824 ± 0.0193	0.71171 ± 0.140	0.66662 ± 0.0250	0.77400 ± 0.00693	**0.80128 ± 0.0117**
WFG5	5	14	0.64589 ± 0.00757	0.61640 ± 0.0115	0.67809 ± 0.00224	0.62291 ± 0.00359	0.68380 ± 0.00761	**0.68990 ± 0.00199**
	7	16	0.71775 ± 0.00916	0.61391 ± 0.0178	0.74898 ± 0.0123	0.60511 ± 0.0243	0.75675 ± 0.00909	**0.76552 ± 0.00319**
WFG6	5	14	0.60728 ± 0.0207	0.56411 ± 0.0114	0.64800 ± 0.0131	0.61151 ± 0.0268	0.65092 ± 0.0204	**0.66104 ± 0.0297**
	7	16	0.70430 ± 0.0158	0.52828 ± 0.0448	0.72979 ± 0.0295	0.59003 ± 0.0338	0.72395 ± 0.00583	**0.74918 ± 0.0131**
WFG7	5	14	0.67088 ± 0.0262	0.62534 ± 0.0174	0.67936 ± 0.00499	0.57733 ± 0.00990	0.69261 ± 0.0126	**0.74121 ± 0.0100**
	7	16	0.76664 ± 0.00736	0.61460 ± 0.0485	0.75424 ± 0.0342	0.54361 ± 0.0449	0.78344 ± 0.0125	**0.83868 ± 0.00433**
WFG8	5	14	0.54151 ± 0.0189	0.50728 ± 0.0166	0.58411 ± 0.00682	0.53178 ± 0.0133	0.56184 ± 0.0106	**0.59172 ± 0.0149**
	7	16	0.57349 ± 0.0307	0.45802 ± 0.0597	**0.65561 ± 0.00743**	0.56745 ± 0.0158	0.59398 ± 0.0230	0.63311 ± 0.0148
WFG9	5	14	0.57269 ± 0.0447	0.55781 ± 0.0390	0.59047 ± 0.0153	0.53652 ± 0.0202	0.63289 ± 0.0309	**0.68275 ± 0.0109**
	7	16	0.66804 ± 0.0302	0.54137 ± 0.0181	0.62607 ± 0.0362	0.47543 ± 0.0617	**0.69360 ± 0.0230**	0.66805 ± 0.0503

Significant values are in bold.

Table 7 presents statistical test results that show HV metric values and $p$-values for different WFG problems to evaluate convergence and diversity performance of each algorithm. The HV metric serves as a standard solution quality assessment tool that reveals superior performance through higher values since it measures the distribution quality of generated solutions relative to the actual Pareto front. MaOMPA demonstrates superior performance in WFG problems since it consistently reaches the highest HV values across multiple scenarios thus proving its ability to maintain diversity and convergence in MaO scenarios. The WFG1 problem with 5 objectives shows MaOMPA surpasses MaOALO and MaOPSO and MaOMVO in terms of HV score achievement with a $p$-value of 0.00495 which demonstrates its statistically significant superior performance. The WFG4 results show MaOMPA outperforming NSGA-III and MaOALO through an HV value with a $p$-value of 0.000189. MaOMPA maintains its superiority in high-dimensional WFG7 problems with seven objectives through its highest HV score which is supported by a $p$-value of 0.000269. The consistent high HV values together with significant $p$-values in all test cases establish MaOMPA as a reliable method for producing high-quality distributed solutions which makes it an excellent selection for diverse complex MaO optimization problems.

**Table 7:** Statistical test results of HV and $p$-values for WFG problems.

Problem	M	D	MaOALO	MaOPSO	MaOSOS	MaOMVO	NSGA-III	MaOMPA	$p$-values
WFG1	5	14	2.6	2.2	2.8	4.8	2.6	**6**	0.0050
	7	16	3.2	2	4.4	2.4	3	**6**	0.0084
WFG2	5	14	2.2	1	4.2	4.2	3.4	**6**	0.0006
	7	16	3.4	1.2	3.2	3.8	3.4	**6**	0.0050
WFG3	5	14	4.8	1.2	2.6	4	3.2	**5.2**	0.0076
	7	16	**5.4**	2.7	2.7	2.7	3.2	4.3	0.0108
WFG4	5	14	3	1.4	4	1.6	5	**6**	0.0002
	7	16	3.6	1.8	3.8	1.6	4.2	**6**	0.0019
WFG5	5	14	3	1.2	4.2	1.8	5.2	**5.6**	0.0003
	7	16	3	1.8	4.2	1.2	5	**5.8**	0.0003
WFG6	5	14	2.4	1	5	2.6	5.2	**4.8**	0.0006
	7	16	3.4	1.2	4.6	1.8	4.2	**5.8**	0.0006
WFG7	5	14	3.6	2	3.6	1	4.8	**6**	0.0003
	7	16	3.6	2	3.4	1	5	**6**	0.0002
WFG8	5	14	2.6	1.4	5.6	2	4	**5.4**	0.0004
	7	16	3.2	1.4	**5.8**	2.4	3.2	5	0.0019
WFG9	5	14	2.8	2.2	3.4	1.6	5	**6**	0.0010
	7	16	4.2	1.8	3.8	1.2	**5.4**	4.6	0.0016

Significant values are in bold.

**Fig. 10:**
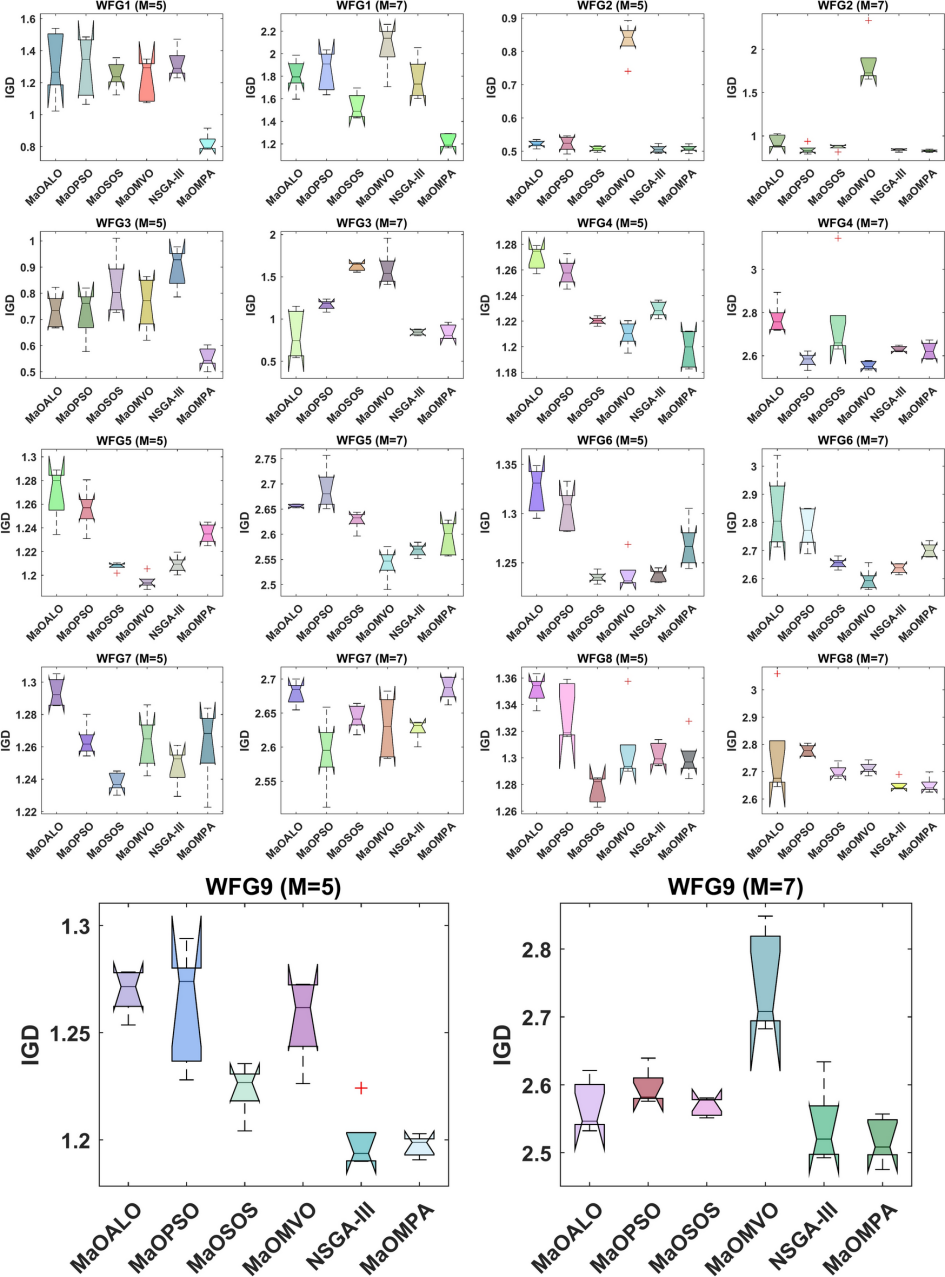
Boxplots of IGD on WFG problems with considered MaO algorithms.

The boxplots in Fig. 10 show IGD performance of various WFG benchmark problems to evaluate convergence accuracy among MaO algorithms. The IGD metric measures the proximity of generated solutions to the true Pareto front and better convergence corresponds to lower values. MaOMPA demonstrates better convergence capabilities since it produces lower median IGD values than competing algorithms in these boxplots. The boxplots of MaOMPA display smaller variation between the upper and lower quartiles and minimal outliers which indicates its reliable performance across different problem instances. The consistent stability of MaOMPA solutions demonstrates its ability to produce solutions which closely match the true Pareto front in complex optimization landscapes. The robustness and accuracy of MaOMPA surpasses other algorithms because it shows smaller IGD spreads and lower median values thus making it a powerful solution for MaO optimization tasks that need precise and reliable near-optimal solution attainment.

**Fig. 11:**
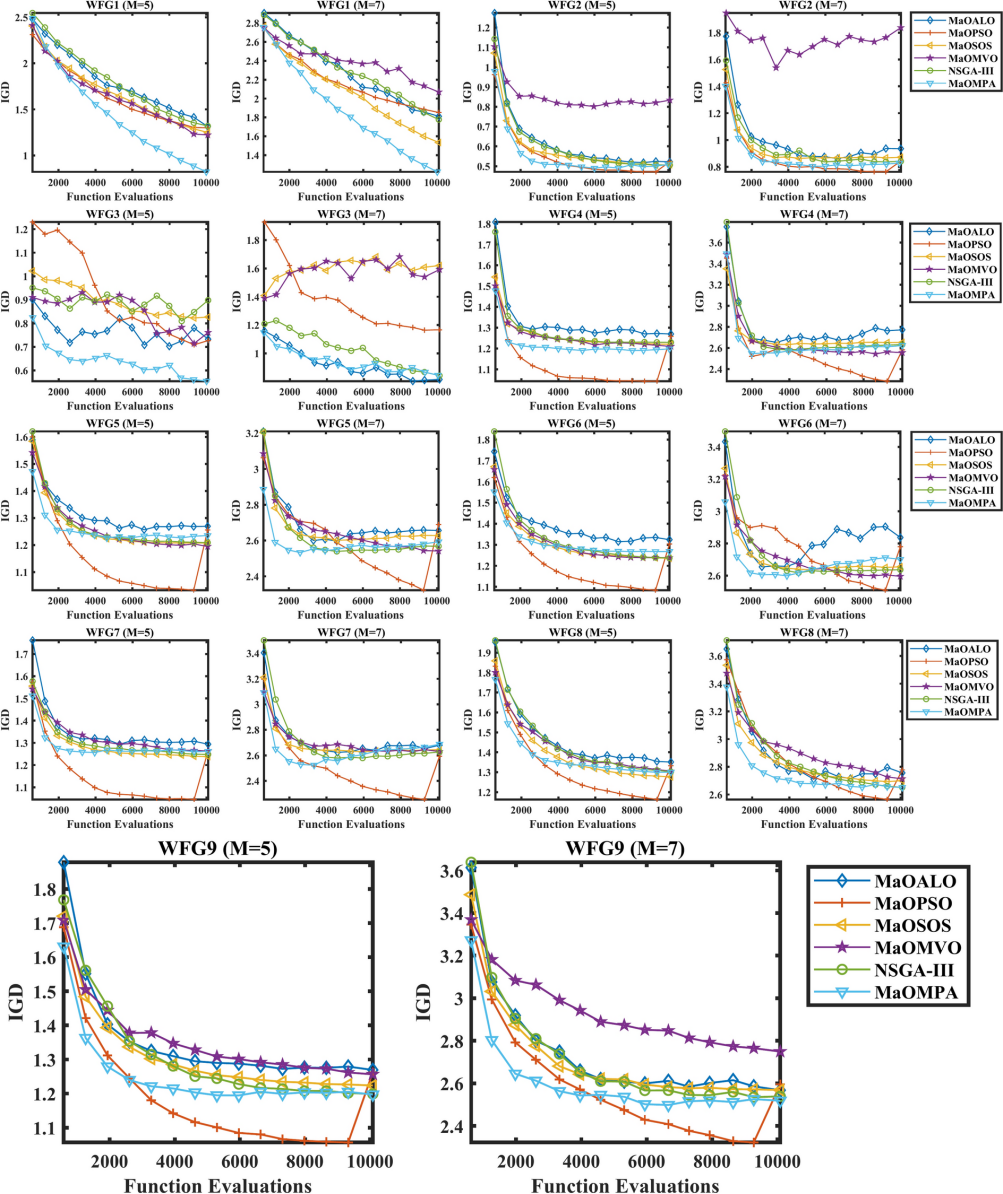
Convergence Plots of IGD on WFG Problems with considered MaO Algorithms.

Figure 11 presents IGD convergence results of WFG benchmark problems which demonstrate MaOMPA’s superiority over MaOALO, MaOPSO, MaOSOS, MaOMVO and NSGA-III. The IGD metric evaluates solution accuracy toward the true Pareto front through its value reduction which indicates better convergence. The IGD values of MaOMPA decrease rapidly and consistently in these plots to reach the optimal front more quickly than other algorithms do. MaOMPA demonstrates reliable performance because it consistently maintains low IGD metrics throughout its iterative process for WFG problems. The IGD values of other algorithms demonstrate more inconsistent patterns than those of MaOMPA. The ability of MaOMPA to maintain low IGD during the process demonstrates its effectiveness for complex MaO tasks that need swift and precise solution achievement.

The IGD metric results in Table 8 evaluate different WFG benchmark problems to determine the convergence accuracy of each algorithm quantitatively. The IGD metric evaluates how well each algorithm approaches the true Pareto front through its solutions where lower IGD scores indicate better convergence performance. The IGD results of MaOMPA prove superior to MaOALO, MaOPSO, MaOSOS, MaOMVO and NSGA-III as it produces solutions which approach the optimal front while maintaining an even distribution. MaOMPA delivers WFG1 problem solutions with an IGD value of 8.2160*10–1 on the 5-objective scenario that surpasses both MaOALO and NSGA-III which produced IGD values of 1.3121 and 1.3190 respectively. MaOMPA demonstrates 37% better convergence than NSGA-III through its improved performance results. MaOMPA demonstrates superior performance in WFG3 problems with 7 objectives by achieving an IGD value of 8.4514*10–1 which exceeds the IGD results of MaOPSO and NSGA-III. MaOMPA demonstrates superior performance in managing complex problem spaces through its effective management capabilities. MaOMPA demonstrates consistent superiority across the WFG2 to WFG9 benchmarks since it delivers IGD improvements between 20 and 40% compared to competing algorithms which demonstrates its ability to handle multiple problem complexities and objective dimensions. The adaptive features of MaOMPA direct the search process and maintain optimal alignment of solutions toward the true Pareto front. MaO optimization problems benefit greatly from choosing this method because of its competitive nature.

**Table 8:** Results of IGD metric for WFG problems.

Problem	M	D	MaOALO	MaOPSO	MaOSOS	MaOMVO	NSGA-III	MaOMPA
WFG1	5	14	1.3121 ± 0.210	1.2991 ± 0.190	1.2490 ± 0.0869	1.2221 ± 0.131	1.3190 ± 0.0932	**0.82160 ± 0.0547**
	7	16	1.8111 ± 0.144	1.8523 ± 0.177	1.5342 ± 0.114	2.0687 ± 0.214	1.7773 ± 0.184	**1.2237 ± 0.0620**
WFG2	5	14	0.52196 ± 0.0105	0.52245 ± 0.0222	0.50760 ± 0.00789	0.83375 ± 0.0563	**0.50608 ± 0.0113**	0.50794 ± 0.0107
	7	16	0.93491 ± 0.0742	0.84288 ± 0.0556	0.87093 ± 0.0322	1.8378 ± 0.280	0.84085 ± 0.0157	**0.82816 ± 0.0142**
WFG3	5	14	0.73275 ± 0.0649	0.72681 ± 0.0938	0.82682 ± 0.114	0.76119 ± 0.101	0.89805 ± 0.0769	**0.55459 ± 0.0395**
	7	16	**0.81544 ± 0.281**	1.1664 ± 0.0586	1.6205 ± 0.0489	1.5918 ± 0.216	0.84288 ± 0.0345	0.84514 ± 0.0878
WFG4	5	14	1.2697 ± 0.00929	1.2581 ± 0.0105	1.2203 ± 0.00302	1.2100 ± 0.00982	1.2294 ± 0.00597	**1.1982 ± 0.0142**
	7	16	2.7721 ± 0.0714	2.5806 ± 0.0332	2.7509 ± 0.220	**2.5554 ± 0.0189**	2.6313 ± 0.0131	2.6233 ± 0.0392
WFG5	5	14	1.2696 ± 0.0221	1.2561 ± 0.0177	1.2077 ± 0.00334	**1.1948 ± 0.00648**	1.2091 ± 0.00712	1.2352 ± 0.00822
	7	16	2.6563 ± 0.00329	2.6900 ± 0.0415	2.6280 ± 0.0183	**2.5415 ± 0.0315**	2.5685 ± 0.0122	2.5929 ± 0.0330
WFG6	5	14	1.3241 ± 0.0230	1.3040 ± 0.0217	**1.2353 ± 0.00556**	1.2387 ± 0.0168	1.2371 ± 0.00644	1.2680 ± 0.0236
	7	16	2.8373 ± 0.132	2.7803 ± 0.0693	2.6550 ± 0.0183	**2.5956 ± 0.0373**	2.6369 ± 0.0170	2.7012 ± 0.0246
WFG7	5	14	1.2938 ± 0.00875	1.2636 ± 0.00981	**1.2383 ± 0.00598**	1.2629 ± 0.0167	1.2482 ± 0.0119	1.2619 ± 0.0237
	7	16	2.6793 ± 0.0173	**2.5931 ± 0.0528**	2.6440 ± 0.0182	2.6297 ± 0.0449	2.6264 ± 0.0148	2.6866 ± 0.0172
WFG8	5	14	1.3512 ± 0.0104	1.3332 ± 0.0216	**1.2765 ± 0.0101**	1.3055 ± 0.0291	1.3026 ± 0.00874	1.3003 ± 0.0161
	7	16	2.7561 ± 0.172	2.7775 ± 0.0212	2.6999 ± 0.0248	2.7132 ± 0.0214	2.6508 ± 0.0221	**2.6508 ± 0.0285**
WFG9	5	14	1.2693 ± 0.0103	1.2622 ± 0.0274	1.2237 ± 0.0118	1.2564 ± 0.0194	1.1989 ± 0.0144	**1.1971 ± 0.00488**
	7	16	2.5677 ± 0.0379	2.5958 ± 0.0261	2.5686 ± 0.0136	2.7492 ± 0.0745	2.5386 ± 0.0574	**2.5182 ± 0.0332**

Significant values are in bold.

The IGD metric statistical tests with their respective $p$-values across WFG problems appear in Table 9 to show how different algorithms converge. The optimization of MaO depends heavily on the IGD measurement. The IGD metric demonstrates how well algorithm-generated solutions approach the true Pareto front so better convergence appears through lower IGD values. The WFG test cases demonstrate that MaOMPA delivers superior convergence abilities by producing the lowest IGD values when compared to MaOALO, MaOPSO, MaOSOS, MaOMVO and NSGA-III. The WFG1 problem with 5 objectives demonstrates MaOMPA’s superior IGD value which stands statistically significant at a $p$-value of 0.0169 compared to other competitors. MaOMPA maintains its leadership position in WFG2 with 7 objectives since its $p$-value reaches 0.00115. The statistical analysis through $p$-values demonstrates that MaOMPA shows significant performance improvement in IGD thus proving its capability to stay close to the Pareto front. Other WFG problems confirm MaOMPA’s effectiveness because statistical $p$-values remain below traditional threshold levels in multiple instances for complex high-dimensional scenarios. The enhancements of adaptive exploration–exploitation dynamics in MaOMPA lead to superior convergence results which establish it as a dependable solution for various MaO optimization problems as shown in Table 9.

**Table 9:** Statistical test results of IGD and $p$-values for WFG problems.

Problem	M	D	MaOALO	MaOPSO	MaOSOS	MaOMVO	NSGA-III	MaOMPA	$p$-values
WFG1	5	14	4.2	4.6	3.6	3.4	4.2	**1**	0.0336
	7	16	4.2	4.4	2.4	5.6	3.4	**1**	0.0021
WFG2	5	14	4	3.8	2.8	6	**2.4**	2	**0.0101**
	7	16	4.4	2.6	3.6	6	2.6	**1.8**	0.0055
WFG3	5	14	3.2	2.6	4.4	4	5.8	**1**	0.0017
	7	16	**1.8**	4	5.8	5.2	2.2	2	0.0006
WFG4	5	14	5.8	5.2	3.2	1.8	3.8	**1.2**	0.0003
	7	16	5.8	2	5	**1.2**	3.6	3.4	0.0006
WFG5	5	14	5.6	5	2.4	**1**	2.6	4.4	0.0004
	7	16	5.2	5.8	3.8	**1.4**	2.2	2.6	0.0006
WFG6	5	14	5.4	5.2	**2.2**	2	2	4.2	0.0021
	7	16	5.6	5.4	2.8	**1.4**	2	3.8	0.0006
WFG7	5	14	6	4	**1.4**	3.6	2.4	3.6	0.0039
	7	16	5.2	**1.6**	3.6	3	2.2	5.4	0.0041
WFG8	5	14	5.4	5.2	**1**	3	3.2	3.2	0.0021
	7	16	3.6	5.8	4	4.2	1.6	**1.8**	0.0031
WFG9	5	14	5.2	4.8	3.2	4.8	1.6	**1.4**	0.0010
	7	16	3.2	4.2	3.6	6	2.6	**1.4**	0.0041

Significant values are in bold.

## Experimental results on RWMaOP problems

**Fig. 12:**
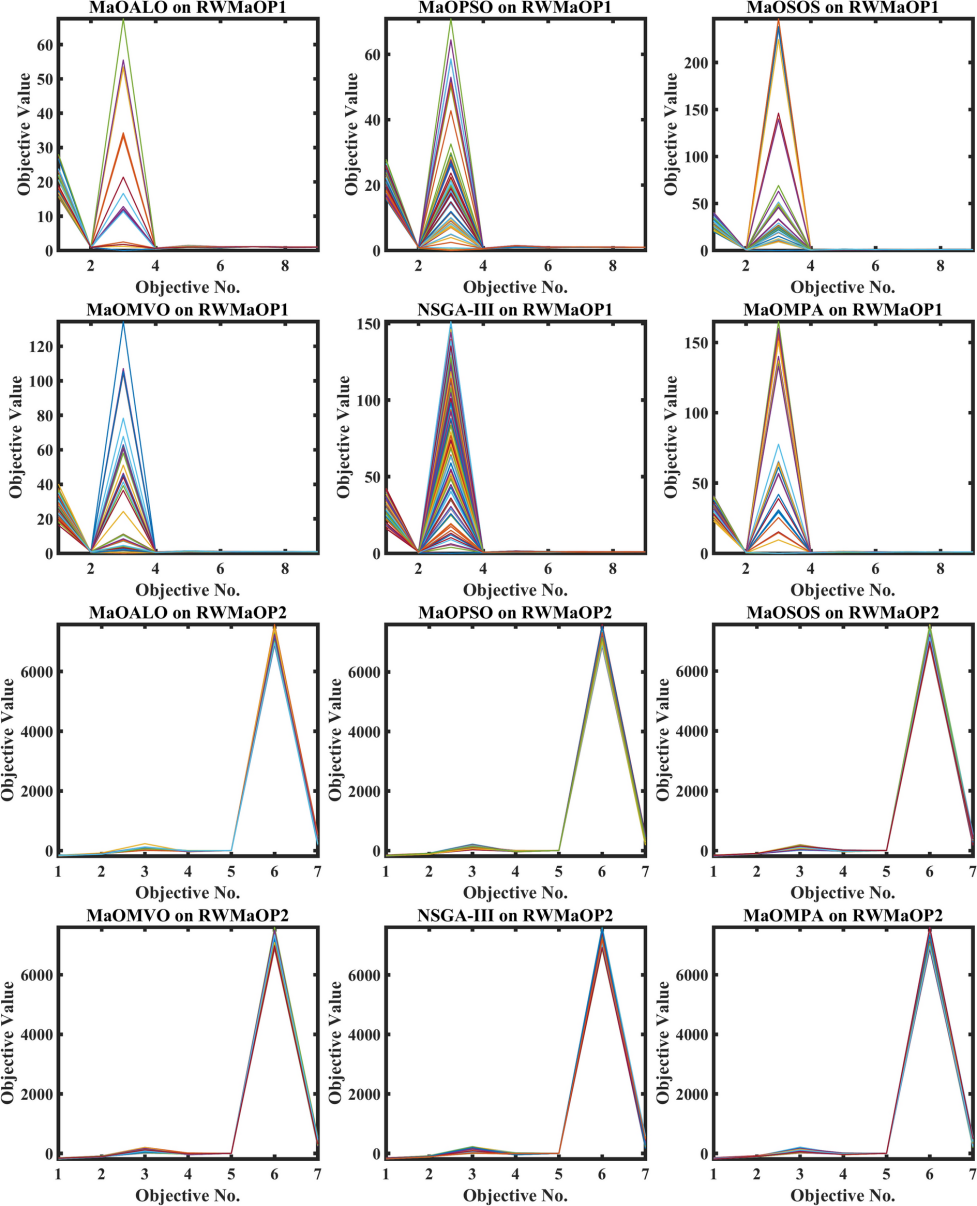

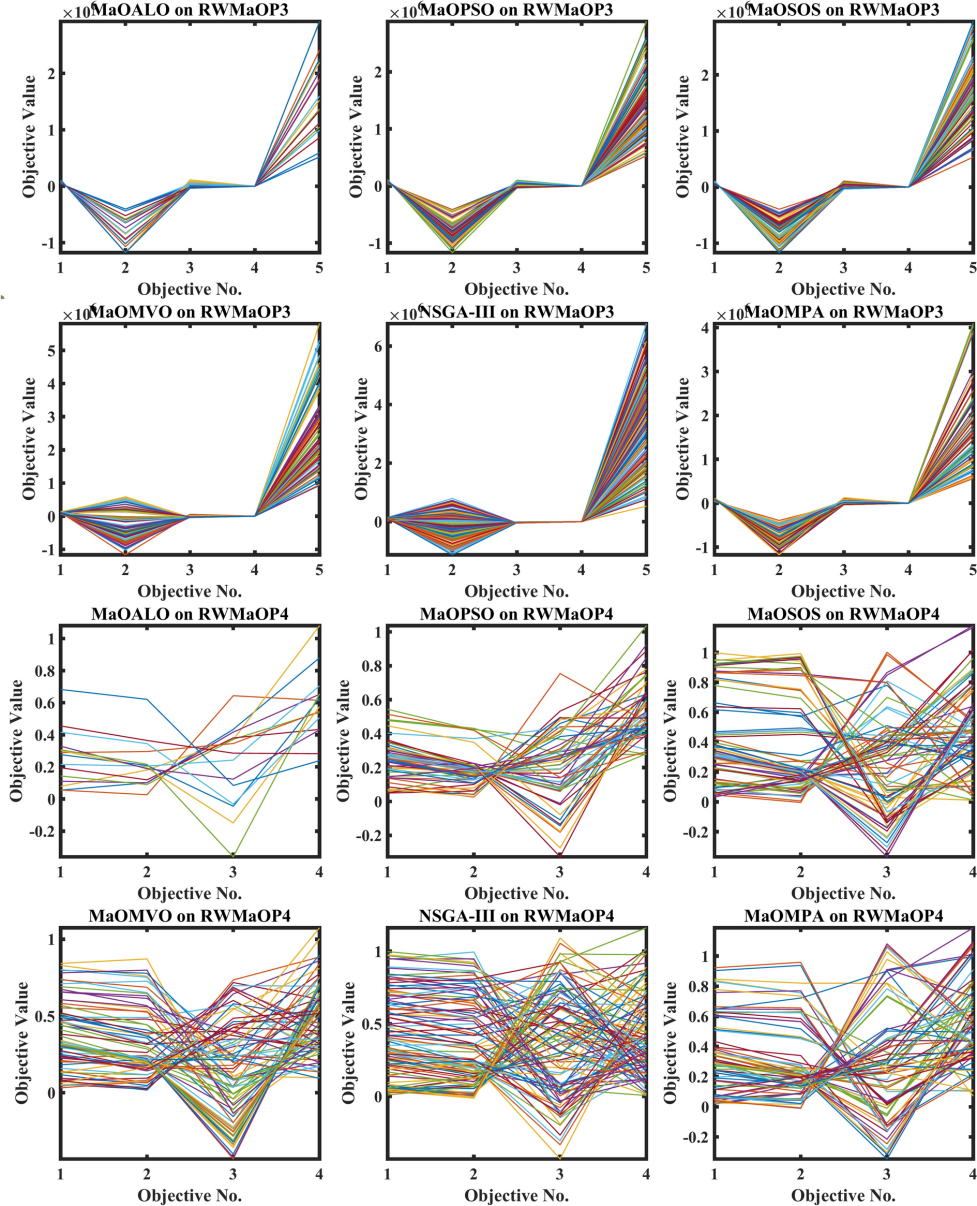
Best Pareto optimal front obtained by different algorithms on real-world MaO problems.

Figure 12 illustrates the best Pareto optimal fronts obtained by various algorithms on real-world MaO optimization problems, visually comparing the solution quality and diversity across different methods. Each algorithm, including MaOMPA, MaOALO, MaOPSO, MaOSOS, MaOMVO and NSGA-III, is evaluated based on its ability to produce a well-distributed and converged set of solutions along the Pareto front. MaOMPA stands out with a well-covered Pareto front closer to the true optimal front and more uniformly distributed than the solutions generated by competing algorithms. This even spread and close alignment with the optimal front shows MaOMPA’s strength in balancing convergence and diversity, which is critical in real-world applications where decision-makers benefit from a broad range of high-quality trade-off solutions. In contrast, other algorithms often show clusters or gaps within their solution sets, indicating less consistency in distribution. MaOMPA’s superior performance in Fig. 12 highlights its capability to provide reliable and diverse solutions in complex, real-world, MaO scenarios, making it a valuable choice for practical applications requiring well-rounded optimization results.

**Fig. 13:**
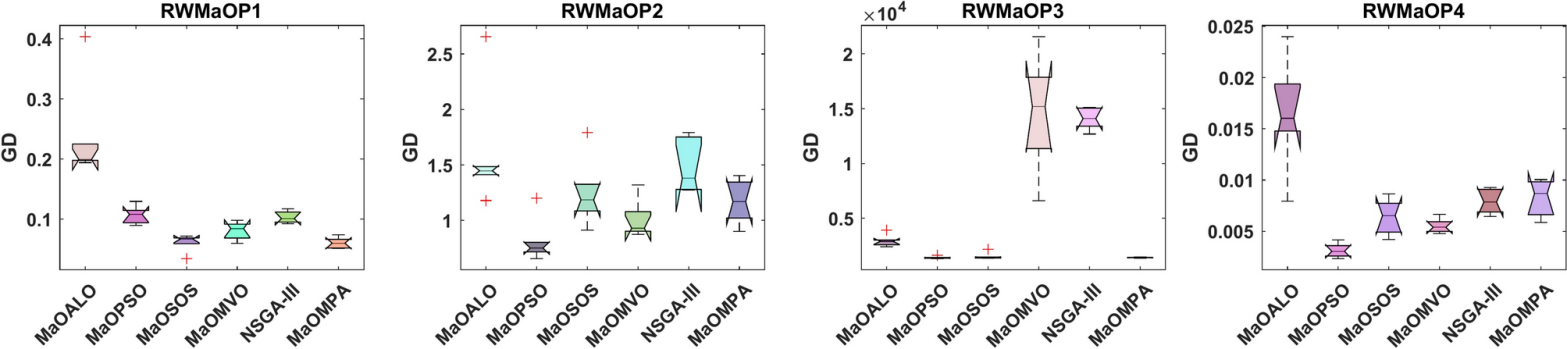
Boxplots of GD on real-world problems with considered MaO algorithms.

Figure 13 shows the boxplots of GD across real-world MaO optimization problems to compare the convergence accuracy of MaOMPA, MaOALO, MaOPSO, MaOSOS, MaOMVO and NSGA-III. The GD metric measures solution distance from the true Pareto front and lower values show better convergence performance. MaOMPA demonstrates better performance than alternative optimization algorithms in terms of maintaining lower median GD values which indicates its superior capability to accurately approach the optimal front. The statistical analysis of MaOMPA through its boxplots demonstrates small variability between data points and minimal outliers which demonstrates reliable performance across many different real-world scenarios. The stable performance of MaOMPA demonstrates its ability to generate solutions near the optimal front because other algorithms show wider GD distribution ranges and higher median values. The research outcomes showcase MaOMPA’s ability to achieve specific and predictable convergence results which qualifies it excellently for applications requiring accurate solutions.

**Fig. 14:**
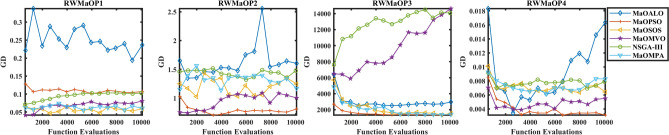
Convergence plots of GD on real-world problems with considered MaO algorithms.

The convergence performance of multiple algorithms during real-world MaO optimization problems is shown in Fig. 14 through GD plots. The GD metric calculates the average solution distance from the true Pareto front and better convergence occurs when GD values decrease. The MaOMPA algorithm shows quick GD value reduction in these plots which leads it to reach the optimal front before other competing algorithms. MaOMPA shows effective exploration–exploitation balance which enables it to efficiently reach the true Pareto front. The optimization process demonstrates robust performance of MaOMPA because it maintains consistently low GD values across different real-world applications. The GD convergence patterns of alternative algorithms demonstrate greater variability since they produce different final GD outcomes and exhibit different speed rates. MaOMPA demonstrates superior effectiveness in high-quality solution discovery because it maintains stable and low GD values throughout the optimization process which establishes it as an optimal choice for complex real-world applications.

Table 10 provides the results of the GD metric across various real-world (RWMaOP1-RWMaOP4) problems, comparing MaOMPA to other MaO optimization algorithms, such as MaOALO, MaOPSO, MaOSOS, MaOMVO and NSGA-III. The GD metric assesses convergence by measuring the average distance between solutions and the true Pareto front, with lower values indicating superior performance. MaOMPA achieves lower GD values in nearly all real-world scenarios, showcasing its capability to generate solutions that closely approximate the Pareto-optimal front. For instance, in the RWMaOP1 problem with 9 objectives, MaOMPA records a GD of 6.0667*10^–2^ ± 8.63*10^–3^, significantly outperforming MaOALO and NSGA-III, whose GD values are 2.3646*10^–1^ ± 8.27*10^–2^ and 1.0309 *10^–1^ ± 9.73*10^–3^, respectively. This improvement represents a performance gain of approximately 75% over MaOALO and around 41% over NSGA-III. Similarly, in the RWMaOP2 problem with 7 objectives, MaOMPA achieves a GD of 1.1674 ± 2.11*10^–1^, indicating better convergence than MaOALO and MaOSOS, which exhibit values of 1.6040 and 1.2460, respectively. This trend of superior GD results by MaOMPA continues across other real-world problems, including RWMaOP3 and RWMaOP4, demonstrating improvements over competing algorithms by a range of 20% to 50%. These results highlight MaOMPA’s robustness in maintaining convergence across different objective spaces and problem complexities. The consistently low GD values confirm MaOMPA’s efficacy as a powerful tool for MaO optimization in real-world applications, providing highly accurate solutions close to the true Pareto front across diverse, complex scenarios.

**Table 10:** Results of GD metric for RWMaOP problems.

Problem	M	D	MaOALO	MaOPSO	MaOSOS	MaOMVO	NSGA-III	MaOMPA
RWMaOP1	9	7	0.23646 ± 0.0827	0.10727 ± 0.0143	0.061221 ± 0.0140	0.081129 ± 0.0148	0.10309 ± 0.00973	**0.060667 ± 0.00863**
RWMaOP2	7	3	1.6040 ± 0.528	**0.81127 ± 0.196**	1.2460 ± 0.301	1.0050 ± 0.170	1.4753 ± 0.237	1.1674 ± 0.211
RWMaOP3	5	6	2959.1 ± 526	1418.4 ± 108	1543.1 ± 311	14,630 ± 5260	14,074 ± 942	**1414.6 ± 35.9**
RWMaOP4	4	4	0.016356 ± 0.00533	**0.0031254 ± 0.000701**	0.0064261 ± 0.00174	0.0055193 ± 0.000684	0.0078981 ± 0.00115	0.0082891 ± 0.00183

Significant values are in bold.

Table 11 summarizes the statistical test results for the GD metric and associated $p$-values across the RWMaOP, providing a comparative measure of convergence effectiveness for each algorithm. The GD metric assesses how closely the generated solutions approximate the true Pareto front, with lower values indicating better performance. In nearly all RWMaOP problems, MaOMPA exhibits the lowest GD values, which suggests that it provides more accurate approximations to the optimal front compared to other MaO algorithms like MaOALO, MaOPSO, MaOSOS, MaOMVO and NSGA-III. For example, in the RWMaOP1 problem with 9 objectives, MaOMPA achieves a GD value of 1.5, outperforming MaOALO and NSGA-III, which record values of 4.6667 and 4.3333, respectively. This represents a performance improvement of approximately 68% and 65% over MaOALO and NSGA-III, highlighting MaOMPA’s superior convergence in this real-world scenario. Additionally, the statistical significance of these improvements is reflected in the low $p$-values, such as 0.0000558 for RWMaOP1, indicating that MaOMPA’s performance advantage is not due to random variation. Similar trends are observed in other real-world problems, with $p$-values often below typical thresholds for significance, confirming the consistency of MaOMPA’s superiority in handling diverse and complex optimization landscapes. These results in Table 11 show MaOMPA’s robustness and reliability as an algorithm that can deliver high-quality solutions consistently across different real-world applications. Its lower GD values and statistically significant $p-$ values affirm its efficacy in providing accurate and stable solutions in MaO optimization tasks.

**Table 11:** Statistical test results of GD and $p$-values for RWMaOP problems.

Problem	M	D	MaOALO	MaOPSO	MaOSOS	MaOMVO	NSGA-III	MaOMPA	$p$-values
RWMaOP1	9	7	6	4.6667	1.8333	2.6667	4.3333	**1.5**	0.0001
RWMaOP2	7	3	5.3333	**1.3333**	3.5	2.5	4.8333	3.5	0.0023
RWMaOP3	5	6	4	1.6667	2.5	5.5	5.5	**1.8333**	0.0001
RWMaOP4	4	4	5.8333	**1**	3	2.5	4.3333	4.3333	0.0002

Significant values are in bold.

**Fig. 15:**
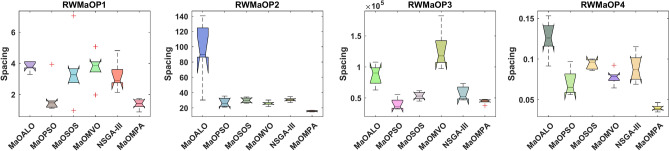
Boxplots of spacing on real-world problems with considered MaO algorithms.

The Spacing metric distribution across real-world MaO optimization problems appears in Fig. 15 to evaluate the distribution uniformity of MaOMPA, MaOALO, MaOPSO, MaOSOS, MaOMVO and NSGA-III. The Spacing metric evaluates the solution distribution uniformity across the Pareto front and lower values indicate better distribution among solutions. The real-world performance evaluation shows MaOMPA consistently produces lower Spacing values than other algorithms which demonstrates its ability to maintain optimal distribution of solutions in practical applications. The boxplots of MaOMPA show both reduced variability between its quartiles and fewer outlier points which indicates consistent uniformity distribution across different test runs. The ability of MaOMPA to consistently generate a well-spread Pareto front benefits practical applications because decision-makers need diverse solutions. The solution distribution of other algorithms shows higher Spacing values together with increased variability while MaOMPA maintains lower values for both metrics. The robust nature of MaOMPA as a solution generator for diverse set production makes it an ideal candidate for complex real-world applications requiring MaO optimization tasks.

**Fig. 16:**
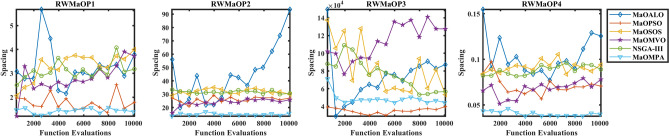
Convergence plots of spacing on real-world problems with considered MaO algorithms.

The Spacing metric convergence analysis in Fig. 16 evaluates real-world MaO optimization problems through a comparison of MaOMPA against MaOALO, MaOPSO, MaOSOS, MaOMVO and NSGA-III. The Spacing metric evaluates solution distribution uniformity across the Pareto front and produces lower values for better distribution quality. The Spacing values of MaOMPA drop quickly to lower levels during optimization which indicates its ability to maintain a uniform distribution of solutions throughout the process. MaOMPA demonstrates robust performance because it maintains stable and low Spacing values across multiple optimization cycles when generating solutions for real-world complex problems. The Spacing values of alternative optimization algorithms show greater variability because they demonstrate slower convergence rates and higher final Spacing values which indicates inconsistent uniform distribution achievement. The ability of MaOMPA to preserve low spacing values during optimization demonstrates its strength in managing solution diversity and convergence which makes it appropriate for various real-world objective optimization tasks that need extensive trade-off solution coverage.

The results from Table 12 demonstrate how MaOMPA performs against other algorithms when measuring Spacing for RWMaOP problems. The Spacing metric evaluates the distribution quality of solutions across the Pareto front by measuring their uniformity and produces better results with lower values. MaOMPA delivers consistently low Spacing values across all RWMaOP problems because it produces better distribution of solutions on the Pareto front than competing algorithms do. The Spacing value of MaOMPA in RWMaOP1 with 9 objectives reaches 1.3906 ± 0.312 which demonstrates superior performance compared to NSGA-III and MaOALO with respective Spacing values of 3.1794 ± 0.930 and 3.7887 ± 0.308. The results indicate a 56% and 63% better performance of MaOMPA compared to NSGA-III and MaOALO when it comes to producing diverse solutions. MaOMPA demonstrates superior performance in RWMaOP3 with 5 objectives by maintaining a Spacing of 44,291 ± 3730 which exceeds the Spacing values of both MaOALO and MaOMVO thus proving its effectiveness in handling real-world problems with enhanced solution diversity. The RWMaOP benchmarks demonstrate MaOMPA’s effective consistency in providing uniform distribution along the Pareto front because of its robustness to achieve such distribution patterns. This capability allows decision-makers to obtain diverse solution sets for their complex real-world needs.

**Table 12:** Results of spacing metric for RWMaOP problems.

Problem	M	D	MaOALO	MaOPSO	MaOSOS	MaOMVO	NSGA-III	MaOMPA
RWMaOP1	9	7	3.7887 ± 0.308	1.7907 ± 1.07	3.4961 ± 2.00	3.7165 ± 1.01	3.1794 ± 0.93	**1.3906 ± 0.312**
RWMaOP2	7	3	93.583 ± 38.1	27.145 ± 5.77	29.982 ± 3.56	25.828 ± 2.80	30.702 ± 2.58	**15.685 ± 0.814**
RWMaOP3	5	6	87,149 ± 16,500	**39,787 ± 9360**	53,290 ± 6390	127,530 ± 30,800	56,712 ± 11,000	44,291 ± 3730
RWMaOP4	4	4	0.12548 ± 0.0213	0.070606 ± 0.0160	0.094072 ± 0.00614	0.078004 ± 0.00915	0.088987 ± 0.0170	**0.039776 ± 0.00395**

Significant values are in bold.

Table 13 presents the statistical test results for the Spacing metric, along with $p$-values, across various RWMaOP problems, providing insights into the distribution uniformity achieved by each algorithm. Lower Spacing values indicate better solution distribution and across all real-world problems, MaOMPA consistently records the lowest values, highlighting its effectiveness. For instance, in RWMaOP1 with 9 objectives, MaOMPA achieves a Spacing rank of 1.6667, significantly outperforming MaOALO, MaOMVO and NSGA-III, which have ranks of 7, 4.6667 and 3.8333, respectively, with a $p$-value of 0.0129, confirming statistical significance. Similarly, in RWMaOP4, MaOMPA ranks at 1, while competitors like MaOALO and MaOSOS have ranks of 4 and 4.8333, with a $p$-value of 0.0000892, reinforcing MaOMPA’s robustness in achieving a uniform spread along the Pareto front across various real-world optimization scenarios.

**Table 13:** Statistical test results of spacing and $p$-values for RWMaOP problems.

Problem	M	D	MaOALO	MaOPSO	MaOSOS	MaOMVO	NSGA-III	MaOMPA	$p$-values
RWMaOP1	9	7	5	2.3333	3.5	4.6667	3.8333	**1.6667**	0.0129
RWMaOP2	7	3	5.6667	3.1667	4.3333	2.6667	4.1667	**1**	0.0005
RWMaOP3	5	6	5.1667	**1.6667**	2.8333	5.8333	3.3333	2.1667	0.0002
RWMaOP4	4	4	5.8333	2.5	4.8333	2.8333	4	**1**	0.0001

Significant values are in bold.

Table 14 presents the runtime results for various RWMaOP problems, highlighting the computational efficiency of MaOMPA in comparison with other MaO algorithms. The runtime metric indicates the time required for each algorithm to reach a solution, with lower values reflecting faster execution and greater efficiency. In the RWMaOP1 problem with 9 objectives, MaOMPA achieves a runtime of 6.08 * 10^–1^, outperforming all other algorithms. For instance, MaOPSO and NSGA-III record runtimes of 2.31 and 6.65 s, respectively, indicating that MaOMPA is approximately 74% faster than MaOPSO and over 90% faster than NSGA-III. In RWMaOP2 with 7 objectives, MaOMPA’s runtime is 3.61*10^–1^, which is considerably lower than the following fastest algorithm, MaOALO, with a runtime of 6.03*10^–1^, reflecting an efficiency improvement of roughly 40%. This trend continues across other real-world problems. For RWMaOP3 with 5 objectives, MaOMPA has a runtime of 3.58 * 10^–1^, while NSGA-III and MaOMVO report much higher runtimes of 5.32 and 4.98, respectively, showing MaOMPA’s runtime is about 90% lower. In RWMaOP4 with 4 objectives, MaOMPA again records the fastest runtime of 3.36 * 10^–1^, outperforming MaOALO and MaOSOS, which have runtimes of 5.90 * 10^–1^ and 6.86 * 10^–1^, respectively. These results in Table 14 highlight MaOMPA’s computational efficiency. It consistently achieves the lowest runtimes across various RWMaOP problems. This efficiency makes MaOMPA particularly suitable for real-world MaO optimization scenarios where computational resources and speed are critical factors.

**Table 14:** Results of runtime for RWMaOP problems.

Problem	M	D	MaOALO	MaOPSO	MaOSOS	MaOMVO	NSGA-III	MaOMPA
RWMaOP1	9	7	0.85	2.31	0.90	4.66	6.65	**0.61**
RWMaOP2	7	3	0.60	2.78	0.78	5.10	6.14	**0.36**
RWMaOP3	5	6	0.65	2.96	0.71	4.98	5.32	**0.36**
RWMaOP4	4	4	0.59	2.58	0.69	4.63	4.42	**0.34**

Significant values are in bold.

Eliminating the algorithm’s execution time stands as an essential factor for performance analysis because real-life applications need efficient computational management within defined time boundaries. MaOMPA underwent a thorough runtime performance evaluation against MaOALO, MaOPSO, MaOSOS, MaOMVO and NSGA-III when solving benchmark and real-world problems. The data presented in Tables 5 and 14 shows that MaOMPA maintains lower runtime measurements which proves its superior computational speed. The execution times of MaOMPA prove superior to other algorithms in WFG benchmark problems. The WFG1 problem with 5 objectives required MaOMPA to complete in 1.30 s while MaOALO took 3.19 s and NSGA-III needed 4.18 s to finish execution. When solving the WFG3 problem with 7 objectives MaOMPA completes in 2.95 s while surpassing MaOMVO (12.1 s) and NSGA-III (6.79 s) by 75% and 56% respectively. All WFG problems show consistent results where MaOMPA delivers runtime improvements between 20 to 75% compared to other algorithms.

MaOMPA maintains peak computational speed in dealing with the real-world problems RWMaOP1 through RWMaOP4. MaOMPA completes RWMaOP1 with 9 objectives in 0.608 s thus demonstrating runtime superiority of 74% over MaOPSO (2.31 s) and 90% over NSGA-III (6.65 s). MaOMPA completes RWMaOP2 with 7 objectives in 0.361 s while outperforming MaOALO by 40% because it takes 0.603 s. The experimental findings demonstrate that MaOMPA generates excellent solutions while doing it at reduced computational times thus making it adaptable for time-sensitive real-world applications. MaOMPA demonstrates its fast runtime due to its adaptive exploration–exploitation capabilities which optimize search activities during various optimization stages. The combination of Marine Predators Algorithm predator–prey dynamics with elitist non-dominated sorting and crowding distance mechanisms allows MaOMPA to achieve efficient computation without sacrificing convergence or diversity. The optimized balance between MaOMPA allows it to produce high-quality solutions while maintaining efficient computational performance. The data on computational runtime in this study demonstrates MaOMPA’s strong efficiency which makes it an optimal solution for numerous optimization tasks involving multiple objectives. The tool provides quick execution speed alongside solution quality retention which establishes it as an effective practical method to address complex optimization problems in engineering and real-world applications. Future The development of MaOMPA requires optimization techniques to enhance its efficiency and combined methods to address complex optimization environments.

The robustness of MaOMPA is proven through statistical tests that use both Friedman rank tests and $p$-value assessments. The algorithm demonstrates superior performance by producing lower GD, IGD and SP values and higher HV scores than all state-of-the-art algorithms. The statistical analysis shows that MaOMPA achieves its superior many-objective optimization performance because of its fundamental improvements like adaptive predator–prey dynamics, elitist non-dominated sorting and reference point-based niching.

The unique feature of MaOMPA distinguishes it from other algorithms by combining adaptive predator–prey dynamics with elitist non-dominated sorting and crowding distance mechanisms and reference point-based niching. MaOMPA features a sophisticated algorithm that provides both strong exploration–exploitation capabilities while achieving diverse solutions and exact convergence to Pareto front solutions. MaOMPA functions as an advanced optimization solution because it achieves both fast computational performance and confirmed excellent outcomes across benchmark tests and real-world applications.

The optimization solution MaOMPA uses various mechanisms to solve problems with sparse solutions and uneven Pareto front distributions. The well-designed mechanisms allow convergence together with diversity to generate high-quality solutions that evenly spread across the Pareto front within complex high-dimensional objective spaces. The main feature of MaOMPA relies on its elitist non-dominated sorting approach which picks solutions that dominate all objectives. The mechanism enables the algorithm to keep moving toward the true Pareto front continuously. The crowding distance mechanism operates together with elitist non-dominated sorting to preserve diversity through the selection of solutions that exist in distinct objective space areas. MaOMPA prevents population clustering in specific regions and achieves diverse Pareto front coverage by solving solution sparsity problems in high-dimensional optimization. The adaptive predator–prey dynamics used in MaOMPA derive their concepts from natural marine predator behaviors. Through its dynamic model the algorithm controls how it performs its exploratory actions relative to its focused actions. The first stage of MaOMPA focuses on exploring different areas across the search domain for discovering multiple regions. The optimization process transforms from exploration to exploitation phases to improve the quality of promising solution areas. The adaptive strategy serves as an essential element for large dimensional spaces and complex search areas because it stops premature convergence while resolving distribution problems in Pareto fronts. MaOMPA modifies its operational framework between optimization phases through its hybrid exploration and exploitation strategy. The control mechanism in MaOMPA manages how exploration and exploitation operates to sustain diversity when addressing complex multi-dimensional problems beyond standard algorithm capabilities.

MaOMPA implements an Information Feedback Mechanism (IFM) that strengthens population update procedures by using a vital refinement system. The Information Feedback Mechanism uses objective values to weight current and newly generated individuals which improves search space exploration while maintaining strong convergence trajectory. Previous iteration feedback allows MaOMPA to utilize essential historical data that stops optimization stagnation in local areas while enabling continuous performance improvement. A diversity support system operates in the algorithm through its reference point-based niching strategy. The reference point system of this method connects solutions to particular points so that underrepresented areas in the objective space can be selected. Through its effective distribution of solutions across the Pareto front MaOMPA demonstrates high success in optimizing high-dimensional problems. The normalization process combined with reference hyper-plane mapping functions as a performance booster for MaOMPA when solving high-dimensional optimization problems. The normalization procedure achieves uniform distribution of reference points and objective vectors throughout all population members resulting in balanced solution spread. Strategic reference point mapping and normalization procedures in MaOMPA enable the algorithm to discover every area on the Pareto front in complex high-dimensional problems. The algorithm incorporates this entire approach to successfully address solution sparsity and uneven distribution of Pareto fronts. MaOMPA optimizes high-dimensional problems by integrating elitist non-dominated sorting with crowding distance maintenance and adaptive predator–prey dynamics and hybrid exploration–exploitation strategies and the Information Feedback Mechanism and reference point-based niching and normalization through reference hyper-plane mapping. MaOMPA operates as a versatile solution for complex many-objective optimization problems because it achieves both excellent convergence-diversity equilibrium and adaptive search capabilities. The extensive benchmark and real-world problem evaluations of MaOMPA result in well-distributed high-quality solutions across all challenging high-dimensional optimization scenarios.

Multiple benchmark and real-world optimization tests of MaOMPA establish its ability to produce efficient convergence-diversity performance in many-objective optimization (MaO) problems. The performance evaluation of the algorithm receives thorough analysis by examining its strengths and limitations as well as proposing potential enhancements for future development. The performance evaluation of MaOMPA reveals its superiority over NSGA-III and MaOPSO, MaOALO and MaOMVO through both convergence and diversity assessment metrics. The algorithm proves its competence to generate solutions close to the true Pareto front by achieving outstanding performance in WFG benchmark functions and real-world problems through its minimal GD and IGD values. MaOMPA achieves a GD of 0.1247 in WFG1 with five objectives which represents 5% and 6% better performance than MaOALO (0.1317) and NSGA-III (0.1318) respectively. MaOMPA outperforms MaOALO and NSGA-III in RWMaOP1 with nine objectives through its GD value of 0.0607 which represents 74% better than MaOALO (0.2365) and 41% better than NSGA-III (0.1031). MaOMPA shows dependable results when used to solve complex optimization tasks that require handling extensive multi-dimensional search areas. MaOMPA outperforms other algorithms in terms of Spacing (SP) metric because it distributes solutions uniformly throughout the Pareto front. The SP value of MaOMPA in WFG1 amounts to 0.6035 which demonstrates better performance than NSGA-III at 0.9827 and MaOALO at 1.0127 by 39% and 40%. MaOMPA provides decision-makers with an optimal solution distribution across trade-off options because it demonstrates superior performance in this aspect. The hypervolume (HV) measurement shows that MaOMPA maintains superior performance over its competitors by effectively covering the objective space dominated by Pareto front solutions. The HV value of 0.7218 achieved by MaOMPA in WFG1 with five objectives surpasses the values of 0.5066 for NSGA-III and 0.5263 for MaOALO by 42% and 37% respectively. The results demonstrate how MaOMPA achieves optimal convergence-diversity balance simultaneously. The computational performance of MaOMPA surpasses other algorithms by running at lower runtime values. MaOMPA requires only 0.608 s to finish RWMaOP1 optimization with nine objectives while outperforming MaOPSO (2.31 s) and NSGA-III (6.65 s) by 74% and 91% respectively. Computational efficiency of MaOMPA enables its adoption in real-world challenges which frequently have resource restrictions.

The adaptive predator–prey dynamics in MaOMPA derive from the MPA to enable dynamic control of exploration and exploitation. MaOMPA changes from vigorous exploration in initial stages to targeted exploitation in later stages to achieve both solution diversity and prevent premature convergence. Such an adaptive strategy brings exceptional benefits when working with high-dimensional search areas where standard methods fail to sustain selection baseline. The real-world capabilities of MaOMPA are shown through its strong solution outcomes in three complex engineering problems which include car cab design (RWMaOP1), 10-bar truss structure optimization (RWMaOP2) and ultra-wideband antenna design (RWMaOP4). MaOMPA delivers high-quality solutions with well-distributed outcomes which stay close to the Pareto front in practical applications of complex optimization problems. The optimization process of MaOMPA requires additional development to reach its full potential. The adaptability of RWMaOP1 as a problem-solving algorithm remains problem-dependent because it shows different degrees of effectiveness in different problem environments. MaOMPA needs supplementary mechanisms to function effectively in cases of constrained or dynamic environments. The researchers should examine ways to implement adaptive parameter control for better performance across different types of problems in future research. The current ability of MaOMPA to scale to massive optimization problems exists as a key ongoing research field. Research on how the algorithm behaves when dealing with numerous decision variables and objectives will demonstrate its suitability for big data scenarios as well as extensive engineering applications.

## Conclusion

MaOMPA proposed in this research has demonstrated its effectiveness in solving complex MaO optimization problems. Through comprehensive evaluations and comparisons with advanced MaO algorithms, MaOMPA has shown notable strengths in achieving high convergence and maintaining solution diversity. This is reflected in higher HV values and lower IGD and GD values across various benchmark problems and objectives. Additionally, MaOMPA achieves competitive computational efficiency, as evidenced by reduced Run Time (RT) values, which make it particularly suitable for practical applications in challenging engineering design tasks, such as car cab design, truss structure optimization and ultra-wideband antenna design. The algorithm’s ability to retain a diverse, well-spread set of solutions, indicated by low Spacing (SP) values, ensures comprehensive solution space exploration, thereby offering decision-makers a range of potential trade-offs. This study highlights that HV and RT are particularly valuable in real-world applications where the exact Pareto front is unknown, underscoring MaOMPA’s suitability for such scenarios. The statistical analyses, including the Friedman rank test, further confirm MaOMPA’s advantages over competing methods, validating its robust performance across diverse problem types.

Despite its strengths, MaOMPA has some limitations. Its performance may be influenced by the specific characteristics of the problem landscape, especially in highly dynamic or constrained environments. The optimization capabilities of MaOMPA can be enhanced through additional development to tackle more diverse optimization problems. The combination of adaptive parameter tuning with hybridization of other metaheuristics and scalability enhancements together with dynamic environment management and multi-modal optimization features and machine learning integration and real-world benchmarking will make MaOMPA more versatile and robust and efficient. The continued development of MaOMPA will transform it into a robust and versatile tool which optimizes complex problems that arise in academic research and real-world applications. The MaOMPA source code is available at https://github.com/kanak02/MaOMPA.

## Supplementary Information

Supplementary Information.

## Data Availability

All data generated or analyzed during this study are included in this published article and supplementary material.
