# Supplementary material for: Adaptive predator prey algorithm for many objective optimization
Source: Sci Rep. 2025 Apr 12;15:12690. doi: 10.1038/s41598-025-96901-y (PMC11993708; doi:10.1038/s41598-025-96901-y)
Supplement: Supplementary file 1 — Supplementary Information. [file 41598_2025_96901_MOESM1_ESM.docx]

# Appendix

## Appendix A: Real World Many-objective engineering design Optimization Problems:

### A.1 RWMaOP1: Car cab design problem [33]

we consider the car cab design problem consisting of 11 decision variables and nine objectives. real world many-objective car cab design optimization problem (RWMaOP1) as follows:

$$\mathrm{minimize}$$

${weight of the car=f}_{1}(x)=1.98+4.9x_{1}+6.67x_{2}+6.98x_{3}+4.01x_{4}+1.78x_{5}+0.00001x_{6}+2.73x_{7}$

$$f_{2}\left( x \right)=Collision Force=1-\left( 1.16-0.3717x_{2}x_{4}-0.00931x_{2}x_{10}-0.484x_{3}x_{9}+0.01343x_{6}x_{10} \right)$$

$f_{3}\left( x \right)=Bumper Displacement=(0.32 -\left( 0.261-0.0159x_{1}x_{2}-0.188x_{1}x_{8}-0.019x_{2}x_{7}+0.0144x_{3}x_{5}+0.8757x_{5}x_{10}+0.08045x_{6}x_{9}+0.00139x_{8}x_{11}+0.00001575x_{10}x_{11} \right))$

$f_{4}\left( x \right)=Rear Seat Displacement=0.32-\left( 0.214+0.00817x_{5} \right.-0.131x_{1}x_{8}-0.0704x_{1}x_{9} +0.03099x_{2}x_{6}-0.018x_{2}x_{7}+0.0208x_{3}x_{8}+0.121x_{3}x_{9}-0.00364x_{5}x_{6}+0.0007715x_{5}x_{10} -0.0005354x_{6}x_{10}+0.00121x_{8}x_{11}\left. +0.00184x_{9}x_{10}-0.018x_{2}x_{2} \right)$

$f_{5}\left( x \right)=Front Seat Displacement=0.32-\left( 0.74-0.61x_{2}-0.163x_{3}x_{8}+0.001232x_{3}x_{10}-0.166x_{7}x_{9}+.227x_{2}x_{2} \right)$

$$f_{6}\left( x \right)=Engine Compartment Displacement=32-\left( \frac{URD*MRD*LRD}{3} \right)$$

$f_{7}\left( x \right)=Roof Displacement=32-\left( 4.72-0.5x-4-0.19x_{2}x_{3}-0.0122x_{4}x_{10}+0.009325x_{6}x_{10}+0.000191x_{11}x_{11} \right)$

$$f_{8}\left( x \right)=Rear Collision=4-\left( 10.58-0.674x_{1}x_{2}-1.95x_{2}x_{8}+.02054x_{3}x_{10}-.0198x_{4}x_{10}+.028x_{6}x_{10} \right)$$

$$f_{9}\left( x \right)=Side Impact=9.9-\left( 16.45-0.489x_{3}x_{7}-0.84x_{5}x_{6}+0.043x_{9}x_{10}-0.0556x_{9}x_{11}-0.000786x_{11}x_{11} \right)$$

Subject to

$$g_{1}(x)=1-\left( 1.16-0.3717x_{2}x_{4}-0.00931x_{2}x_{10}-0.484x_{3}x_{9}+0.01343x_{6}x_{10} \right)\geq0$$

$g_{2}(x)=0.32 -\left( 0.261-0.0159x_{1}x_{2}-0.188x_{1}x_{8}-0.019x_{2}x_{7}+0.0144x_{3}x_{5}+0.8757x_{5}x_{10}+0.08045x_{6}x_{9}+0.00139x_{8}x_{11}+0.00001575x_{10}x_{11} \right)\geq0$

$g_{3}(\boldsymbol{x})=0.32-\left( 0.214+0.00817x_{5} \right.-0.131x_{1}x_{8}-0.0704x_{1}x_{9} +0.03099x_{2}x_{6}-0.018x_{2}x_{7}+0.0208x_{3}x_{8}+0.121x_{3}x_{9}-0.00364x_{5}x_{6}+0.0007715x_{5}x_{10} -0.0005354x_{6}x_{10}+0.00121x_{8}x_{11}\left. +0.00184x_{9}x_{10}-0.018x_{2}x_{2} \right)\geq0$

$g_{4}(x)=0.32-\left( 0.74-0.61x_{2}-0.163x_{3}x_{8}+0.001232x_{3}x_{10}-0.166x_{7}x_{9}+.227x_{2}x_{2} \right)\geq0$

$g_{5}\left( x \right)=32-\left( \frac{URD*MRD*LRD}{3} \right)\geq0$

$$URD=28.98+3.818x_{3}-4.2x_{1}x_{2}+0.0207x_{5}x_{10}+6.63x_{6}x_{9}-7.77x_{7}x_{8}+0.32x_{9}x_{10}$$

$MRD=33.86+2.95x_{3}+0.1792x_{10}-5.057x_{1}x_{2}-11x_{2}x_{8}-0.0215x_{5}x_{10}-9.98x_{7}x_{8}+22x_{8}x_{9}$

$$LRD=46.36-9.9x_{2}-12.9x_{1}x_{8}+0.1107x_{3}x_{10}$$

$g_{6}(x) =32-\left( 4.72-0.5x-4-0.19x_{2}x_{3}-0.0122x_{4}x_{10}+0.009325x_{6}x_{10}+0.000191x_{11}x_{11} \right)\geq0$

$g_{7}(x)=4-\left( 10.58-0.674x_{1}x_{2}-1.95x_{2}x_{8}+.02054x_{3}x_{10}-.0198x_{4}x_{10}+.028x_{6}x_{10} \right)\geq0$

$g_{8}(x)=9.9-\left( 16.45-0.489x_{3}x_{7}-0.84x_{5}x_{6}+0.043x_{9}x_{10}-0.0556x_{9}x_{11}-0.000786x_{11}x_{11} \right)\geq0$

$x_{1}\in[0.5,1.5];x_{2}\in[0.45,1.35];x_{3}\in[0.5,1.5];x_{4}\in[0.5,1.5];x_{5}\in[0.875,2.625];x_{6} \in[0.4,1.2];x_{7}\in[0.4,1.2];$

Where B-Pillar inner ($x_{1}$), B-Pillar reinforcement ($x_{2}$), floor side inner ($x_{3}$), cross members ($x_{4}$), door beam ($x_{5}$), the door beltline reinforcement ($x_{6}$) and roof rail ($x_{7}$).

### A.2 RWMaOP2: 10-bar truss structure problem [34]

A real world many-objective 10-bar truss structure optimization problem (RWMaOP2):

$F_{1}(X)=\text{ mass }=\sum_{i=1}^{m} A_{i}\rho L_{i}$

$F_{2}(X)=\text{ compliance }=\delta^{T}*F$

$F_{3}(X)=\text{ inverse of first natural frequency }=1000000*(\frac{1}{f_{1}})$

$F_{4}(X)=\text{ maximum buckling factor }=max\left( \frac{\left| \sigma_{j}^{comp} \right|}{\sigma_{j}^{cr}} \right)$

Subject to:
Behavior constraints:
$g_{1}(X):$ Stress constraints, $\frac{\max\left( \left| \sigma_{j} \right| \right)-\sigma_{\text{allowable }}}{\sigma_{\text{allowable }}}\leq0$

$g_{2}(X):$ Euler buckling constraints, $\max\left( \frac{\left| \sigma_{j}^{comp} \right|-\sigma_{j}^{cr}}{\sigma_{j}^{\sigma r}} \right)\leq0$, where $\sigma_{j}^{cr}=\frac{kA_{j}E}{L_{j}^{2}}$

Side constraints:
Cross - sectional area constraints, $A_{i}^{\min}\leq A_{i}\leq A_{i}^{\max}$
Mass Density ($\rho$), elastic modulus ($E$) and permittable stress ($\sigma^{\max}$) are assumed as $7850\text{ }kg/m^{3},200GPa$ and $400MPa$ respectively.

### A.3 RWMaOP3: Water and oil repellent fabric development [35]

the water ($f_{1}(\mathbf{x})=-WCA$) and oil ($f_{2}(\mathbf{x})=-OCA$) droplet contact angle; the air permeability $(f_{3}(\mathbf{x})=-AP)$, which measures the airflow through a woven fabric as a comforting property; the crease recovery angle $(f_{4}(\mathbf{x})=-CRA)$, which measures the ability of textiles to recover from creasing; the stiffness ($f_{5}(\mathbf{x})=Stiff$), which is the cotton fabric comfort property; the tear strength ($f_{6}\left( \mathbf{x} \right)=-Tear$) of the finished fabric, which depends on the chemical finishing treatment applied to the fabric; and the tensile strength ($f_{7}\left( \mathbf{x} \right)=-Tensile$) optimization problem (RWMaOP3) as follows:

$$\mathrm{minimize}$$

$f_{1}\left( \mathbf{x} \right)=-WCA=-(-1331.04+1.99\times O\text{-}CPC+0.33\times K\text{-}FEL+17.12\times C\text{-}Temp-0.02\times O\text{-}CPC^{2}-0.05\times C\text{-}Temp^{2}\pm15.33).$

$f_{2}\left( \mathbf{x} \right)=-OCA=-(-4231.14+4.27\times O\text{-}CPC+1.50\times K\text{-}FEL+52.30\times C\text{-}Temp-0.04\times O\text{-}CPC\times K\text{-}FEL-0.04\times O\text{-}CPC^{2}-0.16\times C\text{-}Temp^{2}\pm29.33).$

$f_{3}\left( \mathbf{x} \right)=-AP=-(1766.80-32.32\times O\text{-}CPC-24.56\times K\text{-}FEL-10.48\times C\text{-}Temp+0.24\times O\text{-}CPC\times C\text{-}Temp+0.19\times K\text{-}FEL\times C\text{-}Temp-0.06\times O\text{-}CPC^{2}-0.10\times K\text{-}FEL^{2}\pm413.33).$

$f_{4}\left( \mathbf{x} \right)=-CRA=-(-2342.13-1.556\times O\text{-}CPC+0.77\times K\text{-}FEL+31.14\times C\text{-}Temp+0.03\times O\text{-}CPC^{2}-0.10\times C\text{-}Temp^{2}\pm73.33).$

$f_{5}(\mathbf{x})=Stiff=9.34+0.02\times O\text{-}CPC-0.03\times K\text{-}FEL-0.03\times C\text{-}Temp-0.001\times O\text{-}CPC\times K\text{-}FEL+0.0009\times K\text{-}FEL^{2}\pm0.22.$

$f_{6}\left( \mathbf{x} \right)=-Tear=-(1954.71+14.246\times O\text{-}CPC+5.00\times K\text{-}FEL-4.30\times C\text{-}Temp-0.22\times O\text{-}CPC^{2}-0.33\times K\text{-}FEL^{2}\pm8413.33).$

$f_{7}\left( \mathbf{x} \right)=-Tensile=-(828.16+3.55\times O\text{-}CPC+73.65\times K\text{-}FEL+10.80\times C\text{-}Temp-0.56\times K\text{-}FEL\times C\text{-}Temp+0.20\times K\text{-}FEL^{2}\pm2814.83).$

and $\mathbf{x}=(O-CPC,K-FEL,C-Temp)^{T}$, such that $10\leq O-CPC\leq50$.

### A.4 RWMaOP4: Ultra-wideband antenna design [36]

the voltage standing wave ratio (VSWR) over the passband $(f_{1}(\mathbf{x})=VPVP)$, the VSWR over the WiMAX band $(f_{2}(\mathbf{x})=-VWi)$, the VSWR over the WLAN band $(f_{3}(\mathbf{x})=-VWL)$, the E- and H-planes fidelity factor $(f_{4}(\mathbf{x})=-FF)$ and the maximum gain over the passband ($f_{5}(\mathbf{x})=PG)$ RWMaOP4 is stated as:

$$\mathrm{minimize}$$

$f_{1}(\mathbf{x})=VP=502.94-27.18\times((w_{1}-20.0)/0.5)+43.08\times((l_{1}-20.0)/2.5)+47.75\times(a_{1}-6.0)+32.25\times((b1-5.5)/0.5)+31.67\times(a_{2}-11.0)-36.19\times((w_{1}-20.0)/0.5)\times((w_{2}-2.5)/0.5)-39.44\times((w_{1}-20.0)/0.5)\times(a_{1}-6.0)+57.45\times(a_{1}-6.0)\times((b_{1}-5.5)/0.5).$

$f_{2}\left( \mathbf{x} \right)=-VWi=-(130.53+45.97\times((l_{1}-20.0)/2.5)-52.93\times((w_{1}-20.0)/0.5)-78.93\times(a_{1}-6.0)+79.22\times(a_{2}-11.0)+47.23\times((w_{1}-20.0)/0.5)\times(a_{1}-6.0)-40.61\times((w_{1}-20.0)/0.5)\times(a_{2}-11.0)-50.62\times(a_{1}-6.0)\times(a_{2}-11.0))$.

$f_{3}\left( \mathbf{x} \right)=-VWL=-(203.16-42.75\times((w_{1}-20.0)/0.5)+56.67\times(a_{1}-6.0)+19.88\times((b_{1}-5.5)/0.5)-12.89\times(a_{2}-11.0)-35.09\times(a_{1}-6.0)\times((b_{1}-5.5)/0.5)-22.91\times((b_{1}-5.5)/0.5)\times(a_{2}-11.0)).$

$f_{4}\left( \mathbf{x} \right)=-FF=-(0.76-0.06\times((l_{1}-20.0)/2.5)+0.03\times((l_{2}-2.5)/0.5)+0.02\times(a_{2}-11.0)-0.02\times((b_{2}-6.5)/0.5)-0.03\times((d_{2}-12.0)/0.5)+0.03\times((l_{1}-20.0)/2.5)\times((w_{1}-20.0)/0.5)-0.02\times((l_{1}-20.0)/2.5)\times((l_{2}-2.5)/0.5)+0.02\times((l_{1}-20.0)/2.5)\times((b_{2}-6.5)/0.5)).$

$f_{5}(\mathbf{x})=PG=1.08-0.12\times((l_{1}-20.0)/2.5)-0.26\times((w_{1}-20.0)/0.5)-0.05\times(a_{2}-11.0)-0.12\times((b_{2}-6.5)/0.5)+0.08\times(a_{1}-6.0)\times((b_{2}-6.5)/0.5)+0.07\times(a_{2}-6.0)\times((b_{2}-5.5)/0.5).$

and $\mathbf{x}=\left( a_{1},a_{2},b_{1},b_{2},d_{1},d_{2},l_{1},l_{2},w_{1},w_{2} \right)^{T}$, such that $5\leq a_{1}\leq7$, $10\leq a_{2}\leq12,5\leq b_{1}\leq6,6\leq b_{2}\leq7,3\leq d_{1}\leq4,11.5\leq d_{2}\leq12.5$, $17.5\leq l_{1}\leq22.5,2\leq l_{2}\leq3,17.5\leq w_{1}\leq22.5$ and $2\leq w_{2}\leq3$.
